# Prioritizing Computational Cocrystal Prediction Methods for Experimental Researchers: A Review to Find Efficient, Cost-Effective, and User-Friendly Approaches

**DOI:** 10.3390/ijms252212045

**Published:** 2024-11-09

**Authors:** Beáta Lemli, Szilárd Pál, Ala’ Salem, Aleksandar Széchenyi

**Affiliations:** 1 Institute of Pharmaceutical Technology and Biopharmacy, Faculty of Pharmacy, University of Pécs, Rókus u. 2, H-7624 Pécs, Hungary; pal.szilard@gytk.pte.hu (S.P.); szechenyi.aleksandar@gytk.pte.hu (A.S.); 2 Green Chemistry Research Group, János Szentágothai Research Centre, University of Pécs, Ifjúság útja 20, H-7624 Pécs, Hungary; 3 Department of Pharmacy, Faculty of Health, Science, Social Care and Education, Kingston University, Penrhyn Road, Kingston upon Thames, Surrey, London KT1 2EE, UK; a.salem@kingston.ac.uk

**Keywords:** pharmaceutical cocrystals, drug formulation enhancement, computational cocrystal prediction, in silico cocrystal prediction methods, rational cocrystal design

## Abstract

Pharmaceutical cocrystals offer a versatile approach to enhancing the properties of drug compounds, making them an important tool in drug formulation and development by improving the therapeutic performance and patient experience of pharmaceutical products. The prediction of cocrystals involves using computational and theoretical methods to identify potential cocrystal formers and understand the interactions between the active pharmaceutical ingredient and coformers. This process aims to predict whether two or more molecules can form a stable cocrystal structure before performing experimental synthesis, thus saving time and resources. In this review, the commonly used cocrystal prediction methods are first overviewed and then evaluated based on three criteria: efficiency, cost-effectiveness, and user-friendliness. Based on these considerations, we suggest to experimental researchers without strong computational experiences which methods and tools should be tested as a first step in the workflow of rational design of cocrystals. However, the optimal choice depends on specific needs and resources, and combining methods from different categories can be a more powerful approach.

## 1. Introduction

Cocrystal formation is a promising approach to enhancing the properties of drugs, particularly solubility, stability, and bioavailability [1,2,3,4]. The selection of a suitable conformer next to the active pharmaceutical ingredient (API) is critical in designing pharmaceutical cocrystals, which can improve the performance of drug substances by modifying their physicochemical properties. Conventional methods for cocrystal screening, such as solvent evaporation [5], solution crystallization [6,7], dry [8], liquid-assisted grinding [9], and melt crystallization [10], and others, have been foundational in the discovery and development of new cocrystals. The techniques mentioned have been helpful in the development of new pharmaceutical cocrystal forms with enhanced properties such as solubility, stability, and bioavailability. However, as practical as they may be, these approaches require extensive laboratory work and are trial-and-error-based, often yielding inefficiencies. Combining conventional methods with novel computational and high-throughput technologies can improve researchers’ cocrystal design and development efficiency. The prediction of cocrystals is a multifaceted approach that combines computational and theoretical methods to forecast the formation and stability of cocrystals as part of the workflow of rational cocrystal design [11,12,13]. This predictive capability allows researchers to focus on the most promising candidates for experimental synthesis, ultimately enhancing the efficiency of drug development processes.

**Figure 1:**
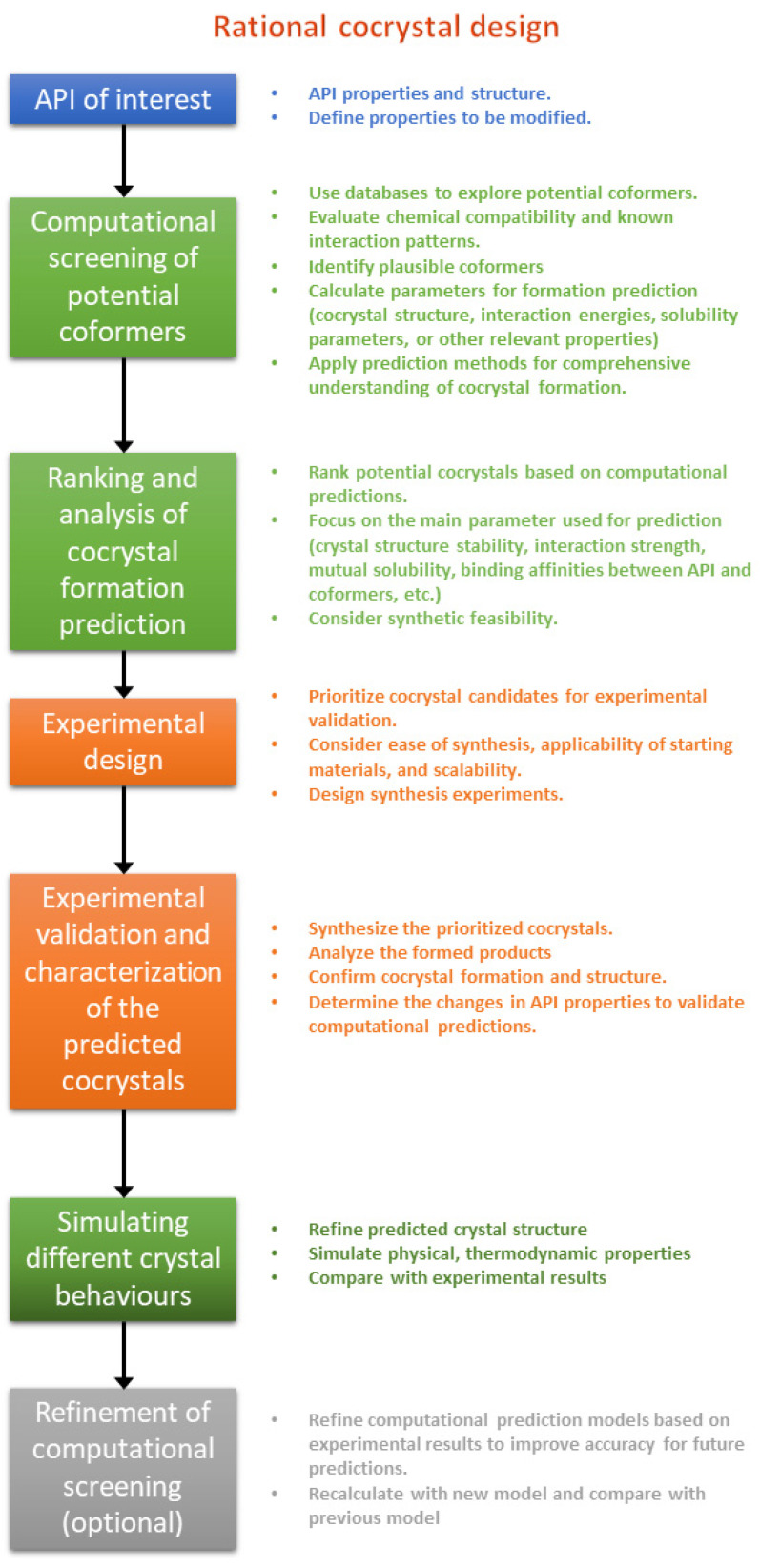
The workflow of rational cocrystal design.

Steps in the workflow of rational cocrystal design are as follows (Figure 1):

1. API of interest: Select an API and clearly define the desired properties of the cocrystal (e.g., improved solubility, stability, and tableting characteristics).

2. Computational screening of potential coformers: Identify potential coformers with complementary functionalities that could enhance the desired characteristics based on chemical compatibility and known interaction patterns. Utilize databases like ZINC [14,15], PubChem [16], and the Cambridge Structural Database (CSD) [17,18] to explore potential coformers based on chemical properties, known interactions, and supramolecular synthons (recurring motifs in crystal structures). Use one or more prediction methods to gain a comprehensive understanding of potential cocrystal behavior with calculating interaction energies, solubility parameters, and other relevant properties.

3. Ranking and analysis of predicted potential cocrystals: Rank the potential cocrystals based on computational prediction of stability and interaction strength. Analyze the results to identify potential cocrystals with favorable interaction energies and binding affinities between APIs and coformers, improved solubility or stability compared to the original API, and synthetic feasibility based on factors like functional group compatibility.

4. Experimental design: Based on the computational prediction and analysis, prioritize the most promising cocrystal candidates for experimental validation, considering factors like ease of synthesis, availability of starting materials, and potential scalability for larger-scale production. Well-defined experiments for cocrystal synthesis can be designed, including solvent selection, crystallization conditions, and purification techniques.

5. Experimental validation and characterization of the predicted cocrystal: Synthesize the prioritized cocrystal candidates using the designed experimental procedures. Then, various characterization techniques like X-ray diffraction (XRD), single-crystal structure analysis (SCXRD), solid-state nuclear magnetic resonance (ssNMR), and spectroscopic methods (e.g., FTIR and Raman) can be employed to confirm the cocrystal formation and determine its structure. Measuring the desired properties (e.g., solubility and stability) of the synthesized cocrystal validates the computational predictions and assesses its potential application.

6. Simulating different crystal behaviors: To gain a deeper understanding of cocrystal behavior, predict or refine the predicted cocrystal structure. Simulate various physical and thermodynamic properties and compare with experimental results.

7. Refinement of the computational screening (optional): Based on the experimental results, refine the computational models (if applicable) for improved accuracy in future predictions.

This workflow emphasizes a data-driven approach that integrates computational screening with well-designed experimental validation. By combining these methods, researchers can efficiently discover and develop cocrystals with desired properties for various applications.

**Figure 2:**
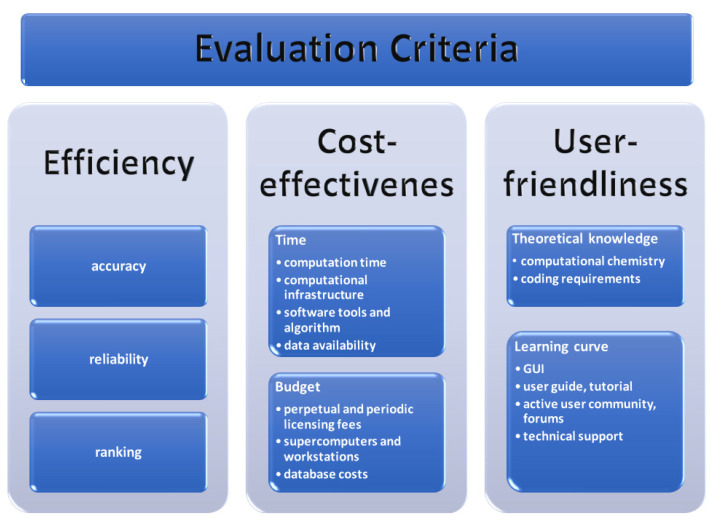
Evaluation criteria: efficiency, cost-effectiveness, and user-friendliness.

Objectives of the present review are to evaluate and prioritize prediction methods based on user-friendliness, efficiency, and cost-effectiveness (Figure 2). The user-friendliness and cost-effectiveness are easily evaluated on an objective basis, but the evaluation of effectiveness is more complex. Primarily, it considers the accuracy and reliability of the predictions, but the amount of information obtained by the method and the time to reach the first results have to be considered as well. Which leaves some space for subjective evaluation of efficiency. The field of pharmaceutical research is increasingly reliant on in silico computational methods for cocrystal formation prediction. While numerous reviews exist [11,13], summarizing the available tools and approaches, none have specifically focused on aiding experimental researchers new to these methods. This study bridges that gap by guiding researchers unfamiliar with computational methods in selecting the most suitable approach for their specific needs. By focusing on methods offering effective predictions with minimal learning curve, time, and financial investment, this study aims to facilitate efficient and informed experimental design for cocrystal discovery.

## 2. Overview of Cocrystal Formation Prediction Methods

The aim of this summary is to assist experimental scientists in making informed choices by highlighting practical applications rather than delving deeply into theoretical foundations and methods. We focus on how the tools can be used effectively rather than on the specific equations solved by the programs in the background. To avoid the black-box effect, we provide a minimal theoretical background, ensuring that users have enough understanding to trust and interpret the results. By offering sufficient theoretical insight without overwhelming the reader, we aim to bridge the gap between complex computational methods and practical experimental needs.

**Figure 3:**
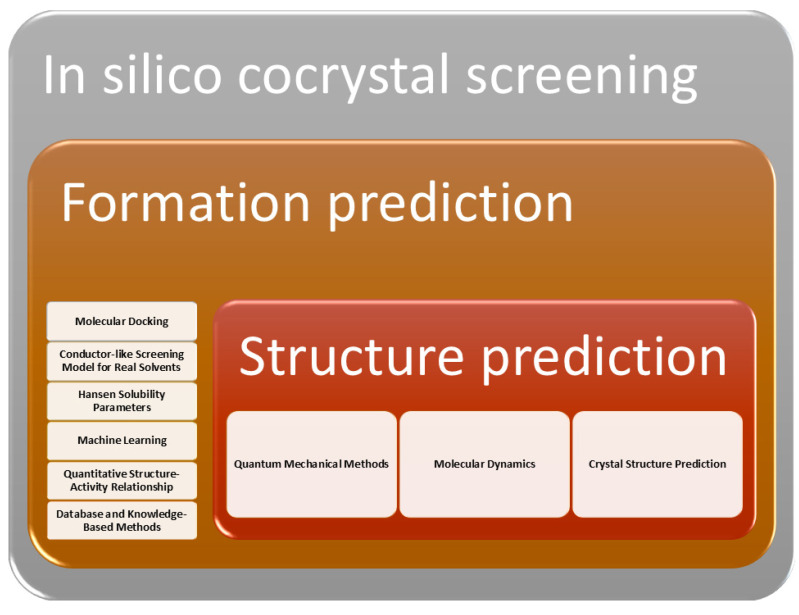
In silico cocrystal screening methods for predicting formation probability and crystal structure.

Many methods and tools are now available to predict cocrystal formations, with some offering insights into not only the probability of formation but also the potential crystal structure of the resulting cocrystals (Figure 3). In this section, we will overview the most commonly used methods, briefly summarizing how cocrystal formations can be predicted using a given method and what software packages and tools are available for it. There is overlap between the categories, and in many cases, they need to be used together. Nevertheless, this division allows us to analyze the available tools according to the criteria of efficiency, cost-effectiveness, and user-friendliness.

### 2.1. Quantum Mechanical Methods

Quantum mechanical (QM) methods provide the underlying theory for understanding the forces between atoms and molecules [19]. QM calculations are used to calculate the electronic structure of the individual molecules (e.g., API and coformer) that form the potential cocrystal [20]. This information provides insights into the reactivity and the ability of molecules to form specific interactions (hydrogen bonding and electrostatics) with another molecule. Furthermore, QM calculations help develop models for intermolecular interactions between the cocrystal components. These models account for factors like electrostatic interactions (based on atomic charges), hydrogen bonding, and dispersion forces [21,22]. The accuracy of these models is crucial for reliable cocrystal prediction [23]. QM calculations can be used to evaluate the interaction energy between API and conformer packed together in the crystal lattice, providing a preliminary assessment of the stability of the potential cocrystal structure [21,22]. While QM offers valuable insights, it is often computationally expensive for complex systems. Simulations might not fully capture all the complexities of real-world cocrystal formation, like kinetic factors or solvent effects. To address these limitations, QM calculations are often combined with other methods like lattice energy minimization or machine learning (ML) to improve efficiency and accuracy [24].

**Figure 4:**
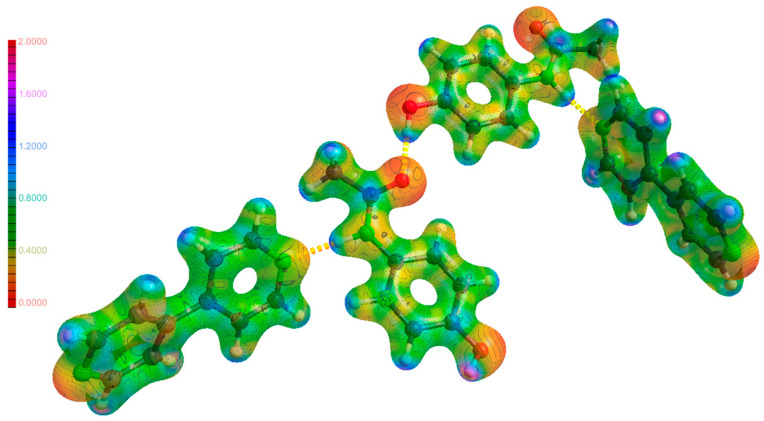
The MEP map of paracetamol–4,4′-bipyridine cocrystal Reprinted/adapted with permission from Ref. [27]. Copyrigth 2018, copyright MDPI, Basel, Switzerland.

QM calculations specifically use methods like density functional theory (DFT) to calculate the electronic structure of molecules and predict interaction energies [25]. Molecular Electrostatic Potential (MEP) calculations analyze the electrostatic potential distribution around molecules to predict interaction sites and complementarity between the API and conformer (Figure 4) [26,27]. This helps in understanding the likelihood of forming stable cocrystals. Periodic DFT calculations offer a more accurate approach to modeling crystal structures compared to in vacuo calculations. Unlike in vacuo methods, which treat molecules in isolation, periodic DFT accounts for the periodic nature of solids, enabling the modeling of long-range interactions between atoms across unit cells. This is crucial for considering intermolecular forces like van der Waals interactions, which significantly influence crystal structures and properties. In contrast, in vacuo calculations often fail to accurately represent these interactions, leading to less reliable predictions. By minimizing edge effects through periodic boundary conditions, periodic DFT provides a more realistic representation of the electronic structure, geometry, and stability of crystals, leading to more accurate predictions of their physical properties [28]. Furthermore, phonon calculations, as achieved through density functional perturbation theory (DFPT), are indeed critical for calculating Gibbs free energy within periodic DFT frameworks by incorporating lattice vibrations and entropy contributions. This approach provides high accuracy in thermodynamic property predictions, which is essential for determining stability and phase transitions in crystalline materials. However, phonon calculations are computationally intensive and require significantly longer processing times, especially for complex systems, making a trade-off between accuracy and computational cost unavoidable [28].

There are several relevant QM programs or tools that utilize the principles of QM to perform calculations on molecules and materials. Gaussian is a commercially available popular software package for performing various electronic structure calculations using quantum chemistry methods like DFT [29]. It is widely used for calculations on molecules and small clusters [30]. CASTEP [31] is a widely used software package for first-principles calculations in materials science, available under a free license for academic users. It employs DFT with a plane-wave basis set to calculate the electronic properties of a variety of materials, including crystalline solids. Vienna Ab initio Simulation Package (VASP) is a powerful software package designed for performing electronic structure calculations on periodic systems, particularly solids and surfaces [32]. It utilizes plane-wave basis sets and pseudopotentials to efficiently handle complex systems. QuantumESPRESSO is an open-source software package known for its capabilities in electronic structure calculations for periodic systems [33]. It offers a modular design and allows for a high degree of customization for specific needs. BIOVIA Materials Studio DMol^3^ is another versatile modeling program based on DFT that predicts chemical processes and material properties across gas, solution, and solid phases, making it valuable in fields such as chemistry, pharmaceuticals, and materials science [34]. The commercially available Schrödinger suite offers a wide range of tools for various tasks in molecular modeling, simulation, and property prediction [35]. Its two modules, namely, Jaguar [36] and MacroModel [37], can be valuable tools within a computational workflow for cocrystal prediction. Jaguar offers high-level electronic structure calculations for accurate interaction energy analysis, while MacroModel is useful for initial structure preparation and basic energy minimizations. OCTOPUS is a powerful computational chemistry program performing electronic structure calculations at the quantum mechanical level [38]. It is a valuable tool for refining and analyzing the predicted crystal structures, offering a deeper understanding of the electronic interactions within the cocrystal.

### 2.2. Molecular Docking and Molecular Dynamics

Molecular docking and molecular dynamics (MDs) utilize pre-calculated data or classical mechanics approaches based on the principles of QM used for molecular simulations [39,40]. Molecular docking simulates the binding of the API and coformer to predict their ability to form a stable cocrystal [41]. Simulating the docking poses provides insights into potential binding modes within the cocrystal structure. Docking programs simulate how the API molecule and potential coformers might interact with each other at the molecular level. They predict the binding site (where the coformer interacts with the API) and the binding pose (the conformation of the coformer in the complex). This information helps researchers identify coformers with favorable interactions that could potentially lead to a stable cocrystal. Many docking programs offer scoring functions that estimate the binding affinity (strength of interaction) between the docked molecules [42]. This can help assess the favorability of a particular cocrystal arrangement. Thus, docking tools can be used to virtually screen a large database of potential coformers for a specific API, prioritizing promising coformers with the help of docking scores researchers can for further evaluation [43]. This helps identify promising candidates for cocrystallization based on their predicted binding interactions. A brief overview of some popular programs is followed.

AutoDock and AutoDock Vina (Vina) are popular open-source docking programs known for their user-friendliness and speed [44,45]. GOLD is a widely used docking program known for its genetic algorithm approach to exploring docking poses [46]. GOLD (Genetic Optimization for Ligand Docking) is commercially available from the Cambridge Crystallographic Data Centre (CCDC) [47]. Schrödinger Suite is a comprehensive software suite that includes Glide for docking as well as tools for structure preparation, virtual screening, and property prediction [48]. As mentioned earlier, this suite is not freely available for academic users; it is commercial software with licensing fees. The University of California, San Francisco (UCSF) maintains the DOCK program, an academic docking program known for its speed and ease of use [49]. The best choice of docking program depends on your specific needs, considering factors like budget, desired level of accuracy, and user-friendliness. Noted here is that although primarily designed for protein–ligand and protein–protein docking, these tools are now widely used beyond protein–ligand docking, including cocrystal formation predictions [41,43], applications such as predicting host–guest interactions, and other non-covalent binding processes [50,51].

MD simulates the dynamic behavior of API–coformer pairs over time to assess the stability of the predicted cocrystal [52,53]. MD simulations are not the typical first-choice method for directly predicting cocrystal structures. This is because simulating the entire crystallization process with full atomistic detail is computationally expensive and time-consuming. However, MD simulations become valuable after the potential cocrystal structures have been identified using other methods like lattice energy minimization or docking simulations with the aim of refining docked poses, studying conformational flexibility, and analyzing intermolecular interaction. Desmond [54], GROMACS [55], AMBER [56], and LAMMPS [57] are valuable tools for MD simulations that can be a powerful complement to other cocrystal prediction methods. They offer insights into the dynamics and stability of potential cocrystal structures, aiding in the selection of promising candidates for further investigation.

### 2.3. Crystal Structure Prediction and Lattice Energy Minimization

Crystal structure prediction (CSP) generates possible crystal structures with the arrangement of atoms (crystal structure) of a material based on its chemical formula [58]. The generated structures are ranked based on lattice energies. It identifies the most stable cocrystal forms by comparing the energies of different possible configurations, and the most stable structure is often the one with the minimum energy. Further approaches within CSP support cocrystal prediction [59]. The evolutionary algorithms mimic natural selection to iteratively improve candidate structures, or the random sampling involves generating a large number of random structures and evaluating their stability.

The lattice energy minimization method is a computational approach used to predict cocrystal structures. It relies on the principle that cocrystals form when the overall lattice energy (the energy holding the crystal structure together) is minimized. Calculates and minimizes lattice energy to predict the most stable crystal based on the core principle: The method explores different possible packing arrangements of the cocrystal components and calculates the lattice energy for each arrangement. The arrangement with the lowest lattice energy is considered the most stable and the predicted cocrystal structure. Thus, it essentially tackles CSP by minimizing an energy function.

CSP is a critical area in materials science and pharmaceuticals, and several software programs have been developed to aid in this process. An overview of various software options for CSP will be discussed; at the same time, it is impossible to list all the available tools because new software and advancements are continuously emerging in the field of CSP.

DMACRYS software developed at the University College London is free of cost for academic scientists in the United Kingdom (UK) and commercially available for non-UK academists and for the industry, with the possibility of further developing and contributing with third-party enhancements [60]. Even though workflows and suite examples help to train and use the program, DMACRYS is not user-friendly software; strong computational chemistry knowledge and other QM programs (e.g., Gaussian) are required to simulate possible crystal structures.

CALYPSO (Crystal Structure Analysis by Particle Swarm Optimization) employs particle swarm optimization techniques for predicting crystal structures [61]. This technique simulates the collective movement of a swarm, where individual particles learn from each other and converge toward optimal positions. In this particle swarm optimization (PSO) algorithm [62], through a series of iterations, the swarm of structures explores the potential energy landscape, with each particle adjusting its position based on the energy of its neighbors. This collaborative approach efficiently can identify low-energy crystal structures. CALYPSO can predict energetically stable or metastable structures based on the chemical composition of the compound. This feature allows researchers to explore various structural possibilities without needing extensive prior knowledge of the crystallography of the compound. CALYPSO is currently interfaced with several major computational chemistry and materials science codes, including VASP [32], CASTEP [31], QuantumESPRESSO [33], GULP [63], and CP2K [64]. This interfacing allows users to leverage the strengths of these established programs while utilizing the efficient structure prediction capabilities of CALYPSO. Additionally, the software can be interfaced with other total energy codes upon user request, providing flexibility for specific research needs. CALYPSO is freely distributed for academic use and comes with thorough documentation, which helps users understand and effectively apply the program to their research projects.

XtalOpt is a free and open-source evolutionary algorithm designed for crystal structure prediction [65]. It performs multi-objective global optimization using properties calculated by external codes to discover novel (meta)stable phases of functional materials. The graphical user interface allows users to define parameters, choose queue systems, and select optimizers for the search process. Multi-objective search functionality enables users to add and optimize multiple properties simultaneously with total enthalpy. The progress table provides continuous updates on optimization time, enthalpy, cell volume, space group, and structure ancestry. XtalOpt supports various queue systems such as SLURM [66], PBS [67], and LSF [68], and optimizers like VASP [32], GULP [63], and CASTEP [31]. The interactive plot tool offers real-time visualization and analysis during the search. The open-source nature and compatibility with multiple external codes of XtalOpt make it a valuable tool for materials science researchers.

AIRSS (Ab Initio Random Structure Searching) utilizes a stochastic approach to explore the potential energy landscape of crystal structures [69]. Sensible random packing arrangements are generated, adhering to realistic density and atomic separation constraints. These structures can optionally incorporate prior crystallographic or chemical information to guide the search. This strategy aims for a broad and unbiased sampling of the configurational space. Subsequent ab initio calculations, often employing DFT, evaluate the energy of each generated structure. By identifying the structures with the lowest energy, AIRSS efficiently locates the most stable crystal configuration for the molecule of interest. The open-source AIRSS package (GPL2 license) requires a Unix-like environment for compilation. Windows users can leverage the Linux Subsystem for Windows. AIRSS seamlessly integrates with CASTEP [31] (available free for academic users and can be purchased through BIOVIA Materials Studio), a leading DFT-based total energy code for material properties. Script modifications for alternative DFT codes are feasible, with provided examples.

Beyond traditional crystal structure prediction, USPEX demonstrates versatility by extending its capabilities to diverse material systems [70]. It efficiently predicts structures for nanoparticles, polymers, surfaces, interfaces, and 2D crystals, showcasing its ability to handle various dimensionalities. Notably, USPEX excels in handling complex molecular crystals, including those with flexible molecules. Furthermore, it offers a fully non-empirical approach, predicting stable chemical compositions and corresponding crystal structures solely from elemental names. USPEX surpasses a purely energetic search by additionally identifying a range of robust metastable structures. Its flexibility extends to various simulation types and the incorporation of prior knowledge. The applicability of USPEX is broad, encompassing the discovery of low-energy metastable phases, stable nanoparticle structures, surface reconstructions, and organic crystal packing arrangements. Notably, USPEX can guide the search for materials with desired physical properties, including mechanical and electronic characteristics. The core of USPEX lies in an efficient evolutionary algorithm; however, it offers flexibility by allowing alternative methods like random sampling, metadynamics, and corrected PSO algorithms. USPEX seamlessly interfaces with a multitude of DFT and classical codes, including VASP [32], GULP [63], QuantumESPRESSO [33], CP2K [64], CASTEP [31], and LAMMPS [57], demonstrating its extensive compatibility. The STM4 toolkit [71] can be used to visualize the results.

PyXtal is an open-source Python library for generating random crystal structures [72]. This versatile tool caters to diverse materials, enabling the creation of atomic and molecular crystals (0-3D) with support for specific symmetries and Wyckoff positions. Integration with Pymatgen [73] and ASE [74] allows import/export of various file formats (e.g., cif). PyXtal offers functionalities for XRD analysis, structural manipulation, and geometry optimization (both internal and external methods). Available under the MIT license on GitHub, PyXtal welcomes contributions and user feedback for continuous improvement.

The Utrecht Crystal Packer (UPACK) program suite offers a methodology for generating hypothetical crystal structures with low potential energy [75]. This approach leverages a pre-defined molecular force field to evaluate intermolecular interactions and guide the packing process. While UPACK excels at creating diverse candidate structures, it is crucial to recognize that the generated list may not solely represent experimentally realizable phases. More sophisticated programs are necessary for a definitive ranking of these structures based on their energetic favorability. Notably, the UPACK suite is freely available for academic research purposes.

GULP is a versatile program designed for material simulations across dimensionalities (0–3D) [63]. It prioritizes analytical solutions, particularly through lattice dynamics, over molecular dynamics whenever possible. GULP offers a diverse selection of force fields and incorporates analytical derivatives up to at least the second order for most force fields, extending to the third order for many, facilitating efficient energy calculations. This software is freely available to academics with valid university email addresses. Additionally, graphical interfaces for GULP are accessible through popular software suites like BIOVIA Materials Studio [34]. Furthermore, BIOVIA Materials Studio Polymorph Predictor [34] enables the prediction of possible polymorphs of a compound based on its molecular structure, which is essential for understanding variations in properties like bioavailability, solubility, and stability. Polymorphism is significant across industries, as different crystalline forms of a compound can exhibit distinct physical and chemical properties crucial to product performance and safety.

GRACE is a software product developed by Avant-garde Materials Simulation Deutschland GmbH that is used to predict the solid-state properties of compounds [76]. GRACE is a robust workflow with a command-line interface that can be run on HPC Linux clusters.

Lattice energy minimization can be performed with the earlier mentioned QM programs, for example, Gaussian or Quantum ESPRESSO. Gaussian is not directly designed for CSP, but its capabilities can be indirectly applied within a cocrystal prediction workflow, particularly for high-level accuracy calculations. It can be helpful for initial structure optimization and accurate binding energy calculation.

CrystalMaker is another valuable tool for visualizing and exploring potential crystal structures in the CSP process [77]. Researchers can build or import the API or coformer molecule into CrystalMaker to facilitate the visualization of different packing arrangements. Using the visualization tools of CrystalMaker, various space groups and packing motifs can be manually generated and explored. Once a promising packing arrangement is identified, the energy minimization tool of CrystalMaker can refine the structure, enhancing its initial geometry. The predicted crystal structure from CrystalMaker can then be exported for further analysis with other software, including ab initio calculations for more precise energy minimization and stability evaluation.

CrystalExplorer is a cross-platform computational tool designed for the investigation and analysis of intermolecular interactions within molecular crystals [78]. It offers functionalities to visualize and quantify these interactions, particularly through the decorated Hirshfeld surface and its two-dimensional fingerprint. This allows researchers to gain insights into crystal packing arrangements by mapping electrostatic potentials onto the surfaces. Additionally, CrystalExplorer facilitates the quantification of intermolecular interaction energies, aiding in a more comprehensive understanding of the forces governing crystal structure formation.

Noted here, the CCDC Blind Test, organized by the Cambridge Crystallographic Data Centre, is a benchmark event designed to evaluate and compare the effectiveness of crystal structure prediction methods with the primary goal of assessing the accuracy and capabilities of various computational methods for predicting crystal structures of unknown molecules [79].

### 2.4. Semi-Empirical Methods

Semi-empirical methods combine elements of both fundamental theory and experimental data. COSMO-RS (Conductor-like Screening Model for Real Solvents) combines quantum chemical calculations to represent the surface of molecules with empirically derived parameters to account for interactions between molecules [80]. Within the workflow of cocrystal prediction, COSMO-RS allows you to calculate some key properties relevant to the potential success of cocrystal formation. Statistical thermodynamics are used to predict the solubility and miscibility of molecules in solvents, aiding in coformer selection without needing full-blown molecular simulations [81]. Large-scale, predictive assessment of cocrystal formation is possible through calculating the excess enthalpy of mixing between API–coformer, which serves as an indicator of their tendency to cocrystallize [82].

BIOVIA COSMO-RS products [83] like COSMOquick [84] and COSMOtherm [85] can be valuable tools within a cocrystal prediction workflow. COSMOquick allows for rapid screening of a large number of potential coformers by estimating their excess enthalpy of mixing with the API, providing an initial assessment of their propensity for cocrystallization [82,86]. This helps identify promising candidates for further investigation. COSMOtherm can then be used for a more detailed analysis of these shortlisted coformers by evaluating the thermodynamic properties, phase behavior, and molecular interactions to offer a more complete picture of the potential success of a cocrystal [82]. While COSMOtherm excels at comprehensive solubility predictions, it is important to remember that its focus is on thermodynamics. Additional factors like crystal packing or specific intermolecular interactions might also be important for cocrystal formation. By combining the rapid screening of COSMOquick with the detailed thermodynamic information from COSMOtherm, researchers can efficiently prioritize potential cocrystal formers for experimental validation.

Developed by Scientific Computing and Modeling (SCM), COSMO-RS seamlessly integrates with other tools within the Amsterdam Modeling Suite (AMS) [87,88]. This includes powerful quantum chemistry software like ADF (Amsterdam Density Functional) [89]. Beyond its core functionality for COSMO-RS calculations, it offers valuable capabilities for thermodynamic analysis relevant to cocrystal prediction. COSMO-RS-ADF can calculate properties like excess enthalpy of mixing, which provides an indication of the energetic favorability of interactions between the API and the coformer in a cocrystal. Additionally, it can predict the solubility of cocrystals, allowing researchers to assess potential benefits compared to the original API. Generating sigma profiles for high-throughput screening within COSMO-RS calculations can be the most time-consuming step. COSMO-RS-ADF also offers a quick method that can be used for fast COSMO-RS thermodynamic property prediction [90].

Furthermore, an open-source alternative to the COSMO-RS model, OpenCOSMO-RS, was introduced by Gerlach and his team [91]. This software offers the functionalities of the model through codebases written in both Python and C++ languages.

Hansen Solubility Parameters (HSPs) [92] is another semi-empirical method that can be used in the workflow of cocrystal prediction. HSP relies on experimental data derived from measurements of solubility in various solvents to establish relationships between the solubility behavior of a solvent and its three key parameters (dispersion, polarity, and hydrogen bonding) to represent different types of intermolecular interactions that influence solubility. However, HSP is not purely based on observation; it incorporates some theoretical concepts from corresponding states theory, particularly the use of geometric mean to estimate interaction energy between molecules. Based on this combination, HSP estimates the compatibility of the API and coformer based on their solubility parameters [93]. By comparing the HSP parameters of the API and the coformer, HSP helps estimate their compatibility for cocrystal formation. Molecules with similar HSP values tend to be more miscible (compatible) and potentially form stable cocrystals. HSPiP (Hansen Solubility Parameters in Practice) is a commercially available software package designed to calculate, analyze, and visualize HSPs for various materials [94]. Although numerous databases offer HSP data, the availability and quality of HSP data can vary depending on the database. Some databases may have more comprehensive data than others, and the data may not always be up-to-date.

### 2.5. Machine Learning and Data-Driven Approaches

ML is providing crystal prediction by analyzing vast datasets of known cocrystal structures, their properties, and failed experiments. Algorithms like Support Vector Machines (SVMs) [95,96] and Random Forests (RFs) [97] learn from this data to identify patterns and relationships between the properties of a molecule and its cocrystal structure. This allows researchers to predict the cocrystal structure of new materials based solely on their molecular composition. While these models excel at predicting crystals similar to those used in training, ongoing efforts focus on incorporating more diverse data and advanced techniques to improve generalizability for a wider range of materials.

Various tools and programs can be used for different aspects of ML for cocrystal prediction. Open-source libraries like scikit-learn (Machine Learning in Python) [98] offer implementations of common algorithms for building and applying ML models. Powerful frameworks like TensorFlow (an end-to-end platform for machine learning) [99] and PyTorch (a machine learning library based on the Torch library) [100] can be used for building complex deep learning models for cocrystal prediction. WEKA (Waikato Environment for Knowledge Analysis), another open-source software suite, provides functionalities for data preprocessing, exploration, and implementation of various ML algorithms [101,102]. CrySPY, an open-source Python crystal structure prediction tool (versioned under the MIT License and running on Unix/Linux platforms), empowers researchers to automate materials discovery workflows [103]. It automates structure generation, optimization, energy evaluation, and candidate selection through ML. CrySPY offers various searching algorithms like random search, evolutionary algorithms, and Bayesian optimization with Look Ahead for efficient exploration. ML is employed for intelligent candidate selection, prioritizing structures for further optimization using external software like VASP [32], QuantumESPRESSO [33], OpenMX [104], soiap [105], or LAMMPS [57]. CrySPY supports both atomic and molecular random structures, catering to diverse material systems. This tool empowers researchers to perform crystal structure prediction simulations without requiring in-depth ML expertise.

QSAR, which stands for Quantitative Structure-Activity Relationship, is a powerful computational technique used to predict the biological or physicochemical properties of a molecule based solely on its chemical structure [106]. QSAR is traditionally used for predicting the biological activities of drugs; its principles can also be applied to cocrystal prediction by leveraging the relationship between molecular structure and the potential for cocrystal formation. KNIME Analytics Platform is an open-source data analytics platform that includes nodes specifically designed for QSAR modeling [107]. It has a user-friendly drag-and-drop interface for building and applying QSAR models without extensive coding knowledge. Furthermore, the commercially available KNIME Hub offers a complete platform for data science [108]. The OECD QSAR Toolbox is a software program designed to assess the hazards of chemicals. It is a free tool that reduces animal testing by predicting the toxicity of chemicals before they are produced [109]. Even though the QSAR Toolbox is a versatile platform for building QSAR models, it requires more effort to customize it for cocrystal prediction.

The combination of QSAR and ML offers a powerful approach to cocrystal prediction [110]. QSAR provides the framework and understanding of the structural factors affecting cocrystal formation, while ML algorithms leverage this knowledge to build powerful predictive models.

### 2.6. Database and Knowledge-Based Methods

Virtual screening is a powerful computational technique used in cocrystal discovery to identify potential coformers that could form stable cocrystals with a specific API. Different factors were considered during the screening. Related algorithms can analyze the shapes and sizes of the API and coformer molecules. Ideally, they should complement each other well, allowing for efficient packing in the cocrystal lattice. Furthermore, the presence of functional groups on both API and coformer molecules is crucial. Screening algorithms might look for complementary functional groups that can participate in strong intermolecular interactions (e.g., hydrogen bonding and π–π stacking) necessary for stable cocrystal formation. Additional properties like solubility or hygroscopicity might also be considered during virtual screening.

CSD, the collection of experimentally determined crystal structures, is a powerful resource for researchers in crystal engineering, and it can be used for identifying potential cocrystal formers by looking at structural motifs and interaction patterns [17,18]. The CCDC software suite [47], including ConQuest [111], Mercury [112], and the CSD Python API [113], are essential tools for identifying cocrystal candidates. ConQuest is used to search the CSD for crystal structures and motifs indicative of cocrystal formation. Mercury enables visualization and analysis of crystal packing and existing hydrogen bonding interactions, crucial for understanding potential cocrystal stability. Mercury software offers functionalities beyond just crystal structure visualization [114]. It can be used for cocrystal prediction through a module called “Molecular Complementarity” within the “Co-Crystal Design” menu. This module relies on pre-defined thresholds for calculated molecular descriptors to assess the likelihood of a molecule cocrystallizing with a target molecule. The concept is that molecules with similar properties (shape and polarity) are more likely to form cocrystals. Mercury software acts as a screening tool in cocrystal prediction. By identifying improbable coformers and ranking promising candidates, it helps streamline the experimental process of finding suitable coformers for cocrystallization. The CSD Python API allows for automated, customized searches and statistical analyses of crystallographic data, integrating with ML models for advanced predictions. Researchers use these tools to retrieve data, analyze interactions, and identify promising API–coformer pairs based on structural motifs and predicted interactions. By automating screening and refining criteria, the CCDC tools streamline the discovery and validation of new cocrystal candidates. Overall, these tools provide a comprehensive approach to cocrystal discovery, leveraging extensive crystallographic data and advanced analytical capabilities.

ZINC Database is a free, public database of commercially available chemical compounds for virtual screening [14,15]. PubChem is a freely accessible database maintained by the National Institutes of Health (NIH) that contains information on a large collection of chemical compounds, including small molecules, biomolecules, and synthetic chemicals [16]. DrugBank is a comprehensive bioinformatics and cheminformatics resource that combines detailed drug data with targets, pathways, and clinical trial information [115]. While not specifically designed for cocrystal screening, DrugBank can be a useful resource for identifying potential coformers with known biological activity or properties that might be beneficial for cocrystal design. ChemSpider is a free chemical database containing information on a vast collection of chemical compounds [116]. The data can be filtered based on various molecular properties relevant to cocrystal formation, such as size, shape, hydrogen bond donors/acceptors, or solubility. It offers information on structure, properties, and links to other relevant databases. Crystallography Open Database (COD) is a public database containing crystal structures of organic, inorganic, and metal-organic compounds determined using various experimental techniques [117]. These databases can be valuable resources for informed selection and filtering of potential coformers in cocrystal discovery. By combining them with other tools like molecular docking software, CSP packages, and cheminformatics toolkits, researchers can enhance their virtual screening workflow for cocrystal prediction.

## 3. Exploring Cocrystal Formation Predictions: Case Studies and Challenges

### 3.1. Case Studies for Theoretically Predicted Cocrystals

#### 3.1.1. Unraveling Cocrystal Structures and Interactions: A Quantum Mechanical Perspective

QM methods, particularly DFT calculations, have emerged as powerful tools for investigating cocrystal structures and intermolecular interactions. These methods offer valuable insights along with experimental techniques, providing a deeper understanding of cocrystal formation at the atomic level [21,26,118,119]. For instance, dispersion-corrected plane-wave DFT calculations were used to elucidate the structures and calculate electric field gradient tensors of Xylazine HCl cocrystals, demonstrating good agreement with experimental data [118]. These calculations were performed within the CASTEP module of Materials Studio 2020 using the RPBE functional. The primary aim was to refine the crystal structures obtained from experimental techniques and compute ^35^Cl electric field gradient tensors. The good agreement between the calculated and experimentally measured ^35^Cl electric field gradient tensors validated the accuracy of the refined structures.

DFT calculations can also explore the changes in electronic properties and intermolecular interactions upon cocrystal formation [21,22,26]. For example, a DFT study on the 5-fluorocytosine-ferulic acid cocrystal revealed significant enhancement in hydrogen bond strength compared to the pure drug, potentially explaining the observed decrease in solubility [21]. The calculations focused on frontier molecular orbitals, MEP, and intermolecular interactions. Furthermore, topology analysis based on the theory of atoms in molecules quantified the intermolecular interactions, particularly the strength of hydrogen bonds. The combined experimental and theoretical approach provided valuable insights into the connection between the microscopic structure and macroscopic physicochemical properties of the cocrystal.

However, the accuracy of DFT calculations can be limited by the chosen functional and the complexity of the system. A study investigating various DFT models for predicting cocrystal lattice enthalpy found that simpler systems yielded consistent results across different models, while complex cocrystals, particularly those containing APIs, exhibited significant variations depending on the chosen functional [23]. This highlights the need for further development of DFT methods or exploration of alternative approaches like AI for improved accuracy in predicting complex cocrystals. The comparison of several DFT models interestingly showed that the most computationally expensive model (PBE0-MBD) did not provide a substantial improvement in prediction accuracy compared to a less demanding model (PBE). The performance of two dispersion correction methods, TS and MBD, was found to be comparable. The DFT-based predictions confirmed the thermodynamic favorability of cocrystallization. However, the computationally inexpensive DFTB models exhibited substantial limitations in accurately predicting the lattice enthalpy of complex cocrystals, likely due to their inability to account for dispersion forces [23]. Although not a recent development, the B3LYP functional remains popular due to its well-established accuracy and efficient balance between computational cost and results. In a present study [20], to investigate the intermolecular interactions in cocrystals, the geometries of the isolated molecules at the B3LYP/6-31G** level of theory were optimized using Gaussian16. This optimization allowed them to calculate the MEP of the molecules, which was crucial for identifying the best donor and acceptor sites for hydrogen bonding and other contacts. The calculated MEP values aligned well with the intermolecular interactions observed in the crystal structures determined by XRD. Overall, the DFT studies provided valuable insights into the nature of the intermolecular interactions in the cocrystals, confirming the reliability and utility of the B3LYP functional in such investigations. Parallel with these Minnesota functionals are also used to describe the intermolecular interactions and electronic properties of cocrystals. In a recent study [26], cocrystallization of a poorly soluble drug, telmisartan, was investigated using again a combined approach of computational screening and analysis of crystal structures from the CSD. MEP calculations were performed using ORCA software with the M062X/6–311++G(d,p) level of theory to identify suitable coformers based on their electrostatic complementarity, with telmisartan successfully demonstrating DFT calculations as an effective approach for cocrystal formation prediction.

QM methods offer various valuable insights into the structures of different cocrystals. MEP surfaces, calculated using programs like Gaussian16 [20,119] or ORCA [26], can identify favorable sites for hydrogen bonding and other intermolecular interactions, aiding in cocrystal design. Additionally, tools like the quantum theory of “atoms-in-molecules” (QTAIMs) and non-covalent interaction plot (NCIplot) provide detailed information on the nature and strength of these interactions within the cocrystal [22]. This study [22] utilizes a combined computational approach to analyze the intermolecular interactions governing the crystal packing of a newly synthesized pterostilbene–theophylline cocrystal. DFT calculations quantify the energetic contributions of various interactions, while the QTAIM and NCIplot tools provide detailed information on electron density, bond paths, and the nature (strength and type) of the interactions, respectively. Additionally, MEP surface analysis is employed to understand the π–π stacking interactions arising from the interplay of electron-rich and electron-poor regions of the cocrystal components. Furthermore, the CSD is interrogated to identify other theophylline cocrystals exhibiting similar interactions. Finally, Hirshfeld surface analysis refines the understanding of intermolecular contacts surrounding the included solvent molecules, providing insights into their role in the crystal structure.

Although QM methods offer a powerful suite of tools for investigating cocrystals, their limitations, particularly for complex systems, necessitate a combined approach that integrates computational methods with experimental data and analysis of existing crystal structures from the CSD [23,26,118,119]. This synergy between theory and experiment is crucial for advancing cocrystal design and understanding.

#### 3.1.2. Unveiling the Power of Molecular Dynamics Simulations in Cocrystal Research

MD simulations have emerged as a powerful tool for predicting and understanding the formation and properties of cocrystals. These simulations can reveal the intricate intermolecular interactions and structural features that drive cocrystal formation, providing detailed insights into key factors such as hydrogen bonding tendencies, electrostatic effects, and other non-covalent forces [52,120]. For instance, MD simulations, coupled with DFT calculations, were used to investigate how a negatively charged maleic acid interacts with protonated drug molecules in water [120]. The simulations revealed that these components form stable assemblies through hydrogen bonding. Notably, the maleic acid preferentially interacts with the drug molecules over the self-association of the drug molecules. These findings suggest that the maleic acid plays a key role in dictating the assembly process in solution. This study highlights the importance of considering intramolecular hydrogen bonds when designing force fields for simulations of pharmaceutical cocrystal formation. By modeling these intermolecular interactions, MD can identify the most favorable cocrystal structures and predict the formation of new cocrystals with a high degree of accuracy, marking a successful case of cocrystal formation prediction [52].

Beyond predicting cocrystal formation, MD provides valuable insights into the structural stability and physicochemical properties of cocrystals. This includes understanding the hydrogen bonding patterns, decomposition mechanisms, and the effects of electric fields on cocrystal energetics. Such insights are crucial for the rational design and development of new cocrystal formulations. A study [121] utilized MD simulations to investigate the cocrystal formed between TKX-50 and HMX, an energetic material. By performing MD simulations on supercell models of both TKX-50/HMX and the individual components, the study found that the cocrystal structure exhibited enhanced stability, especially when HMX substituted TKX-50 on specific facets. The analysis revealed that hydrogen bonding and van der Waals forces played crucial roles in cocrystal formation. These findings not only validate the theoretical predictions but also suggest that the TKX-50/HMX cocrystal may have extensive applications in the field of energetic materials.

The ability to probe the energetics and structural features of potential cocrystals has made MD a powerful tool for computational screening. These simulations can rapidly evaluate a large library of coformer candidates, predicting which are most likely to form stable cocrystals with a given drug molecule. For instance, a study [122] investigating the cocrystal potential between CL-20, an energetic material, and TTX utilized MD simulations within Materials Studio software. This in silico approach analyzed the interactions between the molecules and their binding energy, revealing strong interactions that supported the theoretical prediction of cocrystal formation. Furthermore, the simulations predicted improved properties for the cocrystal compared to CL-20 alone. This example highlights the effectiveness of MD simulations in rapidly screening coformers and guiding the design of cocrystals with tailored properties.

MD simulations offer a significant advantage over traditional CSP searches in the realm of cocrystal polymorphism. CSP searches are often limited to identifying low-energy structures, whereas MD simulations can comprehensively explore the free energy landscape of the cocrystal system. This enhanced exploration capability allows MD simulations to uncover hidden polymorphs that might escape detection by conventional methods. Furthermore, MD simulations can simulate transitions between different polymorphs, providing valuable insights into the transformation mechanisms between cocrystal forms. This information is crucial for optimizing the stability and performance of cocrystals. For example, a recent study [53] investigated cocrystal formation between resorcinol and urea. They employed MD simulations with information entropy to identify potential polymorphs. This method, compared to a standard search, effectively navigated the free energy landscape of the system, uncovering new polymorphs stable under specific pressure conditions. This example highlights the effectiveness of MD simulations in not only discovering unseen polymorphs but also understanding the factors influencing their stability.

Finally, MD enables the precise evaluation of physicochemical, biopharmaceutical, and mechanical properties simultaneously, thus enhancing the solid-form diversity of drugs. By leveraging MD to assess hydrogen bonding tendencies, a new computational prediction methodology has been developed, significantly reducing the need for exhaustive experimental study and improving coformer selection success rates [123]. Validated with experimental results from 145 coformers and 6 drugs, this MD-based approach demonstrated high accuracy and efficiency. The success of the methodology in correctly predicting new cocrystals for nitrofurantoin highlights the vital role MD can play in future large-scale coformer screening efforts. Despite the higher computational complexity of MD simulations compared to other methods, their ability to comprehensively explore the free energy landscape and predict hidden polymorphs makes them a valuable tool for cocrystal design and discovery.

#### 3.1.3. Exploring Cocrystal Interactions with Molecular Docking

Docking studies provide an opportunity to model interactions between APIs and potential coformers. Molecular docking was used to identify the most favorable stoichiometric ratio for cocrystal formation [41]. Telmisartan API and maleic acid coformer were docked to identify a favorable stoichiometric ratio to form cocrystals, which was then experimentally validated and shown to have the most favorable impact on drug efficacy. In another study, docking was used to understand the impact of cocrystallization on the functionality of salicylic acid and to see how the binding properties of salicylic acid change when it forms cocrystals with different molecules [124]. GOLD molecular docking software was used to predict the interactions between ritonavir API and L-tyrosine coformer before creating cocrystals, representing yet another successful case of cocrystal formation prediction [43]. This docking analysis aimed to understand the possibility of hydrogen bonding and van der Waals interactions between the molecules, potentially leading to successful cocrystal formation. The software provided scores to rank the favorability of these interactions, with higher scores indicating a better chance of forming a stable cocrystal. Ultimately, the docking results suggested promising interactions between ritonavir and L-tyrosine, which was then confirmed by successful cocrystal synthesis in the wet laboratory experiments. A present study discusses using molecular docking to predict the stability and possible orientation of various bases on the surface of ketoprofen [125]. This is performed to identify if there is a possibility of salt formation between ketoprofen and the three basic excipients (tris, L-lysine, and L-arginine). The potential cocrystal partners for ketoprofen were identified based on their predicted binding affinities and orientations, and then promising candidates were selected for further experimental validation.

#### 3.1.4. Advancing Cocrystal Prediction: A Focus on Crystal Structure Prediction

CSP plays a crucial role in the workflow of cocrystal prediction by offering high accuracy in predicting experimentally determined cocrystal structures. Studies [126,127,128,129] demonstrate the capability of CSP methods to accurately predict cocrystal structures that align well with experimental data for various systems. CSP can be a starting point for designing cocrystals with tailored properties [130]. However, as highlighted in [131], the accuracy of CSP can be limited, evidenced by less accurate predictions for 4,4′-bipyridine, suggesting either limitations in the method or undiscovered forms. Additionally, the 3:1 stoichiometry of the benzotrifuroxan-1,4-dinitrobenzene cocrystal was accurately predicted and subsequently confirmed experimentally; it is important to note that not all predicted cocrystals were successfully obtained experimentally [127].

The selectivity of CSP is instrumental in predicting the formation of the desired cocrystal over other potential polymorphs or competing cocrystals. For instance, a study [59] demonstrates the use of CSP-Lite to identify promising coformers for a triol intermediate, highlighting its ability to select from various candidates. Additionally, another study [132] presents a method for prioritizing cocrystal formation based on the energetic driving force predicted by CSP, aiding in the selection process. The efficiency of CSP methods is also notable, with works such as the study [59] discussing CSP-Lite, a cloud-computing algorithm that enables faster calculations. Furthermore, [132] emphasizes the efficiency gained by reusing databases for rapid screening of coformers.

Sensitivity to parameters is another important aspect of CSP. As illustrated in a present study [131], the potential discrepancy between prediction and experiment for 4,4′-bipyridine suggests that the accuracy of CSP can be affected by parameter sensitivity or missing information. The ability of CSP to handle flexible molecules is demonstrated in [59], which shows the capability of CSP to manage a flexible triol molecule and identify a suitable cocrystal. This study and ref. [131] highlight the importance of considering conformational flexibility for accurate prediction.

CSP also excels in predicting key interactions and properties of cocrystals, such as stability, solubility, and hygroscopicity, by integrating property prediction tools with CSP calculations. Studies [127,128,129] identify dominant intermolecular interactions (e.g., H-bonding and π–π stacking) in predicted cocrystal structures. The ability of CSP to predict specific stoichiometries is evidenced by Ref. [127], which successfully predicts the 3:1 ratio for a benzotrifuroxan-1,4-dinitrobenzene cocrystal.

The design of cocrystals often relies on a combined approach utilizing both experimental and computational methods. The combined application of SCXRD, Gaussian09, and CrystalExplorer17 has enabled a comprehensive understanding of crystal structures and intermolecular interactions, as demonstrated in the study of the L-ascorbic acid–picolinic acid cocrystal [128]. SCXRD provided the initial atomic arrangement, which was then analyzed computationally to elucidate the stability mechanisms of the cocrystal. DFT calculations with Gaussian09 estimated the energetic contributions of individual fragments and molecule pairs, identifying the R22(9) motif as a key stabilizing factor. CrystalExplorer17 further refined this analysis by calculating interaction energies, taking into account nearest neighbors within 3.8 Å, thereby offering a realistic assessment of intermolecular forces. Complementing these findings, another study integrated computational CSP with experimental techniques to investigate ternary molecular ionic cocrystals [129]. CSP methods predicted the structures of all investigated ionic cocrystals, including flexible binary salt complexes and ternary conjugated acid/base systems. Using CrystalPredictor II software, blind structure prediction accurately reproduced the binary salt complex, while Gaussian09 calculations and hypothetical structure searches revealed a range of polymorphs for the ternary system. Periodic dispersion-corrected DFT calculations with VASP estimated the energetic favorability of ionic cocrystal formation compared to physical mixtures, confirming the energetic advantages. This integration of CSP and experimental methods highlights the potential for a combined mechanosynthesis and CSP approach, facilitating the rapid screening and selection of novel functional ionic cocrystals with tailored properties. Combined computational approaches are also significant. For instance, in a present study, the solid-state assembly of 2,2′- and 4,4′-bipyridine with carboxylic acids was investigated [131]. Potential energy surface scans were performed using Gaussian 09 to explore the conformational landscapes of the bipyridine molecules. The CSD served as a reference for analyzing known crystal structures of the components and identifying recurring packing motifs. Subsequently, CSP was carried out using CrystalPredictor v2 software. This generated a multitude of potential crystal packing arrangements for the bipyridine–acid complexes. Gaussian 09 was then re-employed to calculate the conformational energies and distributed multipoles of the predicted structures. To refine the structures and account for intermolecular interactions, crystal optimizations were performed with CASTEP. Subsequently, CrystalExplorer vpo17 was used to perform intermolecular energy calculations and assess the strength of these interactions. Finally, lattice energies were estimated by calculating the energetic contributions of individual molecules within their crystalline phases. The computational predictions demonstrated good agreement with experimental data for 2,2′-bipyridine. However, for 4,4′-bipyridine, the predictions were less accurate. This discrepancy suggests potential limitations in the current modeling approach or the existence of undiscovered crystal forms for 4,4′-bipyridine–acid complexes.

Future applications of the CSP results regarding cocrystals include the rapid identification and experimental validation of novel cocrystals with optimized properties for pharmaceutical and material science applications. A present study [132] estimates the likelihood of successful cocrystal formation between an API and potential coformers by generating lattice energy minimization databases for the pure API and each candidate coformer. These databases predict the most stable cocrystal structures for various API/coformer stoichiometries, with a more negative difference in lattice energies values indicating higher thermodynamic driving forces for cocrystal formation. Reusing these databases allows for the rapid screening of numerous coformers, significantly enhancing the efficiency of the discovery process. Similarly, CSP calculations were pivotal in designing ammonium nitrate cocrystals with tailored properties, serving as yet another successful example of cocrystal formation prediction [130]. The electrostatic potential surfaces of ammonium nitrate and potential coformers were calculated, identifying guest molecules with complementary electrostatic potential distributions to promote strong intermolecular interactions and predicting the lattice structures of these cocrystals by simulating molecular arrangements and minimizing total energy to find the most stable configurations. These CSP calculations provided a valuable foundation for further investigations into the hygroscopicity, stability, sensitivity, and mechanical properties of the designed cocrystals. These results include the rapid identification and experimental validation of novel cocrystals with optimized properties for pharmaceutical and material science applications.

A study [126] developed a novel virtual screening approach for cocrystal prediction using CSP calculations, incorporating crystallinity as a crucial factor. Using dispersion-corrected DFT (DFT-D), the CSP method estimates the lattice energy difference between the drug, coformer, and potential cocrystal, serving as a metric for cocrystal formation propensity. Validated against indomethacin and paracetamol cocrystallization cases from the CSD, CSP-based predictions outperformed established methods like COSMO-RS. These findings advocate for CSP-based virtual screening in pharmaceuticals for accurate and efficient cocrystal design.

Furthermore, an evolutionary algorithm within USPEX software was used to predict cocrystal structures for high-energy molecules using DFT-D [133]. The evolutionary algorithm successfully recovered known structures with experimental conformations, but a more realistic approach is needed for unknown structures. The study showed that the evolutionary algorithm can predict structures within 20 generations even with limited information. Challenges exist for some crystals, and a lower-cost xTB method compromised accuracy. A future hybrid xTB/DFT-D approach is proposed for larger molecules.

Finally, the 7th CSP CCDC Blind Test provided valuable insights into the progress and remaining hurdles in the field of crystal structure prediction. The encouraging results suggest the potential for these methods to become routine tools for materials science research in the near future. The test revealed a significant improvement in the accuracy of crystal structure predictions compared to previous blind tests. A substantial portion of the predicted structures exhibited good agreement with the experimental data, demonstrating the growing maturity of these computational tools. However, the results also highlighted areas for further development. Complex crystal structures with multiple molecules in the unit cell or those involving conformational flexibility proved more challenging to predict accurately.

#### 3.1.5. COSMO-RS: A Valuable Tool for Streamlining the Initial Stages of Cocrystal Development

COSMO-RS has emerged as a powerful tool, streamlining the initial stages of cocrystal development. This computational approach analyzes intermolecular interactions at a molecular level, providing valuable insights for prioritizing promising coformers for experimental validation. COSMO-RS can significantly reduce the number of candidates requiring experimental validation by predicting factors like intermolecular interactions and excess enthalpy of mixing [134,135,136]. By prioritizing promising coformers, COSMO-RS saves time and resources in the initial stages of cocrystal development [137,138]. For instance, in the development of cocrystal forms for the poorly soluble drug abiraterone acetate, COSMOtherm-X, a software suite based on COSMO-RS principles, successfully identified suitable coformers like succinic acid and glutaric acid [139]. This virtual screening significantly reduced the number of candidates requiring experimental evaluations, ultimately leading to the successful creation of new cocrystals with improved properties. Similarly, a study focused on nimesulide, another poorly water-soluble drug, employed COSMOquick, a user-friendly software utilizing COSMO-RS calculations. While COSMOquick indicated a low overall tendency for nimesulide to form cocrystals, it did identify piperazine as a viable candidate [140]. Subsequent experiments confirmed the formation of a cocrystal with piperazine, highlighting the ability of COSMO-RS to pinpoint promising coformers even in challenging cases. A recent study [141] combined nanococrystallization with anti-solvent precipitation to create a soluble form of carvedilol, a β-blocker drug. A key part of their process involved using COSMOquick software to identify the best coformer (tartaric acid) and solvent (acetone) for cocrystallization. This in silico approach, compared to traditional trial-and-error methods, saved time and money in the development process.

Compared to other computational cocrystal prediction methods, COSMO-RS often demonstrates superior accuracy in predicting successful cocrystal formation, highlighting its effectiveness in achieving reliable prediction outcomes [110,137,142,143]. For example, a study designed cocrystals for the drug minoxidil and compared the performance of three different methods, namely, virtual screening CSD, using HSP, and COSMO-RS [142]. While CSD provided valuable insights into suitable coformer functionality, COSMO-RS outperformed HSP in identifying viable cocrystal candidates. The researchers established a specific criteria based on COSMO-RS calculations (calculated excess enthalpy, ΔHex < −2.00 kcal mol^−1^) to shortlist coformers, and this approach successfully led to the creation of new cocrystals of minoxidil with eight aromatic carboxylic acids. Noted here is that another study has also confirmed that although several factors like hydrogen bonding and molecular flexibility were considered, ΔHex values from COSMO-RS served as a preliminary screening tool to identify promising cocrystal candidates for forming multicomponent crystals between 2,4-dichlorophenoxyacetic acid and various N-heterocyclic compounds [144]. Furthermore, COSMO-RS performed the best in predicting successful cocrystal formation of 2-amino-4,6-dimethoxypyrimidine [143]. Based on the predictions from different methods, including COSMO-RS, the researchers were able to identify and experimentally validate 21 new solid phases for 2-amino-4,6-dimethoxypyrimidine, including 10 cocrystals. These examples underline the superior performance of COSMO-RS in predicting successful cocrystal formation compared to other commonly used methods.

While COSMO-RS offers significant advantages, it is important to acknowledge its limitations. In a present study [145] investigating cocrystal formation for the energetic material CL-20, COSMO-RS successfully predicted stable solvents based on factors like enthalpy and cavity volume. However, it struggled to predict cocrystal formation itself, likely due to the entropy contribution [145]. The model excels at predicting intermolecular interactions based on quantum chemistry calculations, but it might not fully account for the role of entropy, another crucial factor influencing cocrystal formation. Additionally, the accuracy of COSMO-RS predictions can be dependent on the specific parameterization used [146]. The investigation of the interactions between sulfa drugs (sulfamethazine and sulfamethizole) and urea in mixtures surprisingly showed that COSMO-RS was not successful in predicting the solid–liquid equilibria phase diagrams [147]. Despite this, the results highlight how COSMO-RS can be used along with other methods to provide a more comprehensive understanding of interactions in pharmaceutical mixtures, even if it does not directly predict phase diagrams [147].

To address these limitations and achieve even more robust predictions, some researchers have explored combining COSMO-RS with other methods. For instance, a study investigating cocrystal design for 2,4-dichlorophenoxyacetic acid employed COSMO-RS along with molecular complementarity (MC) analysis [148]. This combined approach effectively filtered out unsuitable candidates, leading to the identification of new cocrystals for 2,4-dichlorophenoxyacetic acid. At the same time, the multicomponent crystal prediction of the pesticide pymetrozine suggests that COSMO-RS combined with CSD analysis can be an efficient tool for rational cocrystal design [149]. New methods for rational cocrystal design that combine ML with COSMO-RS have also been reported [150,151]. These studies found that combining the predictions from ML and COSMO-RS resulted in a more accurate screening method compared to either approach used alone. This combined method offers a fast and reliable alternative to existing physics-based methods, which can be valuable for pharmaceutical projects with limited time and budget.

#### 3.1.6. The Role of Hansen Solubility Parameters in Cocrystal Prediction

HSPs have been employed as a predictive tool for cocrystal formation by leveraging the principle of “like dissolves like” to estimate the miscibility between an API and potential coformers. This approach utilizes three parameters, namely, dispersive forces, dipole–dipole interactions, and hydrogen bonding, which collectively describe the solubility characteristics of a compound. The effectiveness of HSPs in predicting cocrystal formation has been explored in several studies, each using HSPs to identify promising coformers.

In a study by Li et al. [142], the HSPs of minoxidil and various potential coformers were calculated using the HSPiP software, employing group contribution methods to determine the HSP values and predict miscibility based on the total solubility parameter difference (Δδt). A lower Δδt value suggested higher miscibility and a greater likelihood of cocrystal formation, with a threshold set at Δδt < 7 MP_a_^0.5^ for successful prediction. Despite this, the study found that the COSMO-RS method, which uses ΔHex as a criterion, outperformed the HSP method in predicting successful cocrystal formation. Specifically, the COSMO-RS method achieved perfect prediction with all identified coformers forming cocrystals with minoxidil, whereas the HSP method had a success rate of 65.5%.

Another study explored the use of HSPs for predicting the formation of co-amorphous systems between norfloxacin and various coformers [152]. Here, HSPiP software was used to calculate the HSP values of norfloxacin and 17 coformer candidates. Two approaches were employed as follows: the van Krevelen method, which assesses miscibility based on the difference in solubility parameters (Δδ) with a cutoff of Δδ < 5 MP_a_^0.5^, and the Greenhalgh method, which uses the Δδt with a cutoff of Δδt < 7 MP_a_^0.5^. The study found that the van Krevelen method provided better prediction accuracy for norfloxacin co-amorphous formation. The results suggested that good miscibility, indicated by a lower Δδ value, is essential for forming a stable co-amorphous system.

In another application, HSPs were used to shortlist l-proline as a coformer for ibuprofen in a cocrystal formation study [153]. The workflow involved calculating the total solubility parameters of ibuprofen and potential coformers using the Hoftyzer–van Krevelen method. A key selection criterion was that the difference in total solubility parameter between ibuprofen and l-proline was less than 7 MP_a_^0.5^, indicating good miscibility. Additionally, the selection of l-proline was supported by computational studies using DFT, which predicted increased solvation energy for the ibuprofen-l-proline complex compared to ibuprofen alone, suggesting enhanced solubility in the cocrystal form. These combined analyses (pK_a_ difference, HSPs, and DFT studies) led to the successful preparation and characterization of ibuprofen-l-proline cocrystals using solvent evaporation and melt extrusion techniques, underscoring the importance of the HSP method in achieving these successful outcomes.

Compared to other methods for predicting cocrystal formation, HSPs have demonstrated a lower success rate. Studies have shown that COSMO-RS achieved significantly higher success rates of 82.4% and 84.1%, whereas HSPs only reached 52.2% and 49.0% in the respective studies [137,143]. These findings suggest that COSMO-RS is a more effective tool for the initial screening of coformers in cocrystal formation. Nonetheless, HSPs can still offer valuable insights into cocrystal formation. Another study examined the influence of solvents with different HSPs on the type of cocrystal polymorph that forms [154]. By employing solvents with varying HSPs, researchers were able to generate a new polymorph of a cocrystal. The study highlighted that the ability of the solvent to accept hydrogen bonds had a greater impact on polymorph generation compared to its ability to donate hydrogen bonds. This finding indicates that HSPs can shed light on the interactions between solvents and molecules, which in turn can affect the crystallization process and the resulting crystal structures [154]. Furthermore, recently the potential of using HSPs as a tool to understand how solvent selection can influence cocrystal polymorph generation was also highlighted [155].

While the use of HSPs shows promise for polymorph screening, it is important to recognize their limitations and the need for further research. Understanding how HSPs interact with other factors, such as thermodynamic parameters, is crucial for fully leveraging their potential in predicting cocrystal formation and polymorph screening. Consequently, while HSPs can provide some valuable insights, integrating them with more comprehensive methods like COSMO-RS may enhance the accuracy and reliability of cocrystal formation predictions. Overall, these case studies [137,142,143,152,153,154] demonstrate that while HSPs can be a valuable tool for predicting cocrystal formation, their effectiveness can vary, and they may benefit from being used in conjunction with other computational methods, such as COSMO-RS or DFT calculations, to improve prediction accuracy and reliability in the rational design of cocrystals.

#### 3.1.7. Predicting Cocrystal Success: Leveraging Molecular Features

A novel method for predicting cocrystal formation using a QSAR statistical analysis has been developed, focusing on key molecular features of coformers, such as phenolic acids, that influence their cocrystallization ability with various drugs [156]. By analyzing a dataset of known cocrystal and non-cocrystal pairs, researchers identified 13 important structural descriptors derived from simple SMILES strings, making the method computationally efficient. This approach aims to find coformers effective across a wide range of drugs rather than specific pairings, achieving an estimated 80% accuracy in predicting new cocrystal formations. Another study investigated cocrystal formation using known cocrystal structures from the CSD and employed QSAR to compare various molecular properties of coformers [157]. It found the strongest correlations with shape and polarity rather than the number of hydrogen bond donors and acceptors, suggesting that similar shapes and polarities promote cocrystal formation. These findings highlight the potential of using simple molecular descriptors to develop semiquantitative models for cocrystal prediction. The study also emphasizes the importance of understanding the relationship between these properties and specific supramolecular synthons to improve cocrystal formation success rates. Together, these studies pave the way for developing comprehensive predictive models for cocrystal formation using QSAR.

#### 3.1.8. The Power of Machine Learning in Cocrystal Prediction

ML streamlines cocrystal prediction by automating data analysis and uncovering hidden patterns in large datasets (like CSD). This leads to discovering novel cocrystals and fosters an ever-evolving approach through continuous learning. Moreover, ML excels at handling complex, high-dimensional data, enabling more robust predictions and facilitating large-scale screening efforts. Building an ML model for cocrystal prediction requires data on known successful and unsuccessful pairings. Positive data (known cocrystal formation) typically comes from the CSD, a comprehensive repository of crystal structures [158,159,160,161]. However, the success of these models hinges on acquiring high-quality data encompassing both positive and negative examples [158]. Negative data, representing unlikely cocrystal pairs, poses a challenge. Researchers have addressed this by employing various strategies, including random selection with structural filtering to avoid overly similar molecules [160] or leveraging documented failures from past studies to enrich the negative data pool [161]. These efforts to ensure data quality and balance are essential for developing robust and generalizable ML models that can effectively predict cocrystal formation.

A variety of ML algorithms for cocrystal prediction have been explored, each with its strengths. Popular choices include SVM [162,163], RF [162,163], and Artificial Neural Networks (ANNs) [159,162,163,164]. Notably, XGBoost, a more recent algorithm, has achieved exceptional performance in predicting cocrystal formation [164,165]. Open-source software libraries like scikit-learn provide a versatile toolkit to train and evaluate these ML models in cocrystal discovery [160,162,165].

ML models require rigorous evaluation to ensure their effectiveness and applicability. Researchers utilize various metrics to assess model performance [150,158,159,160,161,162,163,165]. Common metrics include accuracy, ROC-AUC (Receiver Operating Characteristic—Area Under the Curve), precision, and recall. Accuracy reflects the overall proportion of correctly classified cases (true positives and true negatives), while ROC-AUC provides a more robust measure, particularly for imbalanced data, by considering both true positive and false positive rates. Precision and recall offer insights into the ability of the model to identify true positives and avoid false positives. Studies report high accuracy exceeding 80% [159,165] and ROC-AUC values above 0.8 [160], demonstrating the strong classification capabilities of the models. Furthermore, techniques like k-fold cross-validation are employed to assess the generalizability of the model beyond the training data [162,166]. This ensures the model performs well on unseen data, not just the data used for training. Finally, ML models are compared with traditional methods for cocrystal prediction. This comparison aims to show that the ML approach offers superior performance or additional benefits, such as lower computational complexity [160].

Techniques required to understand the reasoning behind their predictions. Feature importance analysis is a crucial tool in achieving this goal. By analyzing feature importance, researchers can identify which molecular descriptors, representing the chemical properties of the molecules, contribute most significantly to the output of the model [162,163,164]. Techniques like shapley additive explanations assign a contribution score to each feature, providing insights into its influence on the prediction of the model [164]. For example, a study on flavonoid cocrystal prediction analysis revealed that descriptors associated with hydrogen bonding were the most impactful features, underlining the critical role of hydrogen bonding in this context [163]. However, interpreting complex models, particularly deep neural networks, remains a challenge [150]. For instance, one study [159] utilized ANNs trained on cocrystal data from the CSD to assess pairs of candidate coformers based on their molecular structures and predict the probability of cocrystal formation. By combining predictions from multiple ANNs, the approach achieved high accuracy, estimated at 80%, even for molecules lacking prior cocrystal information. Notably, the method is applicable to virtually any molecule, including those not yet synthesized, making it valuable for early-stage drug design and optimization within the pharmaceutical industry. Another study [162] used a data-driven machine learning approach combining database virtual screening and QSAR/QSPR analysis to predict cocrystal formation between APIs and coformers. A dataset of successful and unsuccessful cocrystal formations was compiled, and molecular descriptors were generated using Mordred software. Various machine learning models (ANN, SVM, RF, and XGB) were trained with scikit-learn, achieving promising results. Similarly, another approach [165] employed the XGBoost machine learning model from the scikit-learn library, using SMILES strings and RDKit molecular descriptors. This model, trained on data from the CSD and documented non-cocrystal formations, demonstrated exceptional performance with a prediction success rate surpassing 90%. A different study [160] described a machine learning model using the CSD as a source of positive samples and a novel method for generating negative samples via random selection with a filtering step based on structural similarity. PubChem fingerprints represented the molecular structures, and scikit-learn implemented various machine learning algorithms, resulting in a high score on an independent test set and effective experimental validation with captopril. Lastly, a deep forest model [161] was developed using a dataset exceeding 8000 samples from the CSD and documented failures. This model, utilizing ECFP4 and FCFP4 molecular fingerprints, exhibited reduced sensitivity to class imbalance and faster training speed compared to deep learning methods, successfully predicting febuxostat cocrystal formation.

Several studies have utilized large databases, particularly the CSD, to develop predictive models. These extensive databases are used not only for direct identification of potential candidates but also to train and validate ML algorithms that can predict the behavior and interactions of these compounds. By leveraging the vast amounts of data available, ML models can uncover patterns and relationships that may not be immediately apparent through traditional screening methods. This approach enhances the efficiency and accuracy of identifying compounds with desired properties, ultimately accelerating the discovery and development process in fields such as pharmaceuticals and materials science. Although databases primarily serve as starting points for ML models today, database virtual screenings allow us to search for compound groups with specific properties. ML can be very powerful when dealing with large datasets and identifying complex, non-obvious patterns. However, virtual screening with tools like Mercury offers valuable advantages in terms of interpretability, flexibility, and human expertise integration.

#### 3.1.9. Navigating the Cocrystal Landscape: The Virtual Screening Approach

Recent studies have demonstrated the utility of various virtual screening methods and computational tools for predicting cocrystal formation, with a particular focus on CSD. Furthermore, the effectiveness of CCDC software, particularly Mercury, in implementing knowledge-based virtual screening methods for cocrystal design has been highlighted. One key approach involves leveraging MC and hydrogen-bond propensity (HBP) analyses. MC, implemented within Mercury software, assesses the geometric fit between the API and potential coformers based on their shape and polarity descriptors [167,168]. This initial screening helps eliminate sterically incompatible candidates. HBP calculations, also performed in Mercury, evaluate the likelihood of specific hydrogen bond formations between the API and potential coformers [167,168,169]. Hydrogen bonds are crucial for stabilizing cocrystal structures, and HBP analysis helps prioritize coformers with a high propensity for such interactions.

The effectiveness of combining these methods is evident in a study by Ref. [167]. The authors observed only moderate accuracy for individual methods (hydrogen-bond energy (HBE) for successful formations and MC for unsuccessful formations). However, by focusing on the region where both MC and HBP predicted the same outcome, the success rate for cocrystal prediction reached 81%. This emphasizes the importance of a multi-criterion approach for virtual screening. Furthermore, a multicomponent score can also be used to prioritize coformers, resulting in the identification of promising candidates for further experimental evaluation [168].

Another study [170] explored the utility of MC, HBP, and MEP maps for predicting linezolid cocrystal formation. Similar to the previous study, MC analysis in Mercury assessed geometric fit, while HBP calculations evaluated hydrogen bond propensity. MEP maps, generated with external software (Gaussian16), provided insights into intermolecular interaction energies. All three methods demonstrated satisfactory performance, but their strengths differed across the selection process. MC and HBP were recommended for initial screening due to their focus on geometric fit and hydrogen bonding, with the multicomponent score of HBP aiding in prioritizing coformers. MEP maps offered a more energy-based approach for refining the selection, particularly when dealing with a shortlist of similar coformers.

A case study involving caffeine and 4-chlorophenylboronic acid [171] utilized the CSD to identify potential coformers based on structural features and HBP. Mercury software analyzed hydrogen bond patterns, aromatic interactions, and packing similarity of the cocrystal polymorphs, while CSD-Materials and CSD-Particle provided additional insights into the energetics and stability of the cocrystals. This study underscored the value of CCDC tools in identifying potential coformers, predicting interaction types, and analyzing cocrystal structures in detail. By leveraging extensive databases like the CSD and employing sophisticated computational tools such as Mercury, researchers can enhance the efficiency and accuracy of identifying promising cocrystal candidates. Although these studies [167,168,170,171] collectively highlight significant advancements in virtual screening for cocrystal prediction, a recent study compared the effectiveness of a knowledge-based approach for discovering novel pharmaceutical cocrystals to traditional systematic screening and showed that systematic screening is not better than random screening [172]. In this specific work, the CSD and Mercury tools were used to search for known examples of interactions between functional groups of each drug/coformer pair and to estimate the likelihood of obtaining a cocrystal, respectively. Subsequently, experimental cocrystallization screening was performed between each pair of drugs and coformers. Interestingly, the study revealed that systematic screening offered no significant advantage over random screening, missing roughly 25% of successful cocrystal formations. Furthermore, by analyzing intermolecular interactions within known crystal structures, the CSD offers valuable guidance for cocrystal design. A study highlights the use of CSD analysis to predict the prevalence of specific interactions within cocrystals [173]. Employing ConQuest software, the CSD was searched for structures containing pyrazinecarboxamides and perfluorinated alkanes, prioritizing those with verified 3D coordinates. The analysis confirmed expectations, revealing that halogen–aromatic interactions and hydrogen bonds were the dominant forces governing the assembly of these molecules in the cocrystals. This successful prediction again underscores the potential of CSD analysis as a tool for guiding cocrystal design.

The importance of virtual screening using the CSD is further emphasized by a case study on enantiospecific cocrystal formation, underscoring the successful application of this method in predicting cocrystal formation [174]. While performing a cocrystal screening on chiral target compounds, researchers also virtually screened the CSD for relevant entries. The experiment identified 13 novel cocrystal systems, with 11 being enantiospecific. Virtual screening of over 250 chiral cocrystal structures from the CSD revealed a similar trend, with 86% exhibiting enantiospecificity. This highlights the effectiveness of CSD analysis in predicting enantiospecific cocrystal formation. Another example showcasing the utility of the CSD for virtual screening comes from the design of ionic cocrystals, particularly for salts with limited representation in the database [175]. While previously established design principles for neutral cocrystals failed to predict success for ionic cocrystals of ammonium nitrate, a packing coefficient greater than 83.5% was observed consistently among all successful coformers. This observation allowed for the targeted identification of two additional ionic cocrystals. Furthermore, a study demonstrates cocrystal solvate prediction using CSD and Mercury by analyzing solvent propensity for cocrystal solvate formation [176]. The study demonstrates the successful synthesis and characterization of 3,5-dinitrobenzoic acid–acetamide cocrystal solvates. CSD analysis provided valuable insights into solvent selection for cocrystal solvate formation based on the interplay of size, shape, and hydrogen-bonding properties. This information can guide future efforts to design and synthesize novel multicomponent solid forms. Finally, a new web-based application named Cocrystal Pro was developed to prioritize experimental screening for cocrystal discovery [177]. It integrates three in silico predictive tools as follows: HBP, HBE, and MC. HBP analyzes the likelihood of specific hydrogen bond formation between an API and a coformer based on statistics from the CSD. HBE compares the energy of these hydrogen bonds using molecular electrostatic potential surfaces. MC uses five molecular descriptors related to size, shape, and polarity to assess the compatibility between API and coformer.

Rational cocrystal design, a key component of cocrystal development, involves systematically identifying and optimizing cocrystals with desired properties. While computational methods are valuable, they often face limitations in simultaneously predicting both cocrystal formation and structure. Virtual screening, molecular docking, and HSPs are employed to identify promising cocrystal pairs. Thermodynamic calculations, such as those using COSMO-RS, assess the stability of predicted pairs, while QM methods provide accurate intermolecular interaction calculations. CSP methods generate and evaluate potential crystal structures, with lattice energy minimization and molecular dynamics simulations aiding in optimization. While computational prediction approaches can outperform traditional systematic screening and potentially identify cocrystals with fewer experiments, the accuracy of these predictions depends heavily on the chosen methods and tools.

### 3.2. Challenges Faced in Cocrystal Prediction

Cocrystal prediction and development present several significant challenges that emanate from data availability [178], limitations in computational modeling [179,180], the need for robust experimental validation [177], and a comprehensive understanding of both thermodynamic and kinetic factors [181,182]. These challenges underscore the inherent complexity of predicting and synthesizing cocrystals, which are crucial for enhancing the properties of pharmaceuticals and other materials.

One of the primary hurdles is the limited and sometimes inconsistent data about known cocrystals [178,183]. Computational models heavily rely on accurate and comprehensive datasets for training. Incomplete or inaccurate data can lead to misleading predictions and hinder the ability of the models to generalize to new systems [177]. The complexity of intermolecular interactions, not fully captured in existing databases, adds another layer of difficulty in building robust prediction models [184].

The interpretability of complex ML models poses another significant challenge. The lack of interpretability, often referred to as the “black box” nature, makes it difficult to understand how predictions are made [185]. This can undermine researcher confidence and hinder the identification of potential biases or limitations within the model.

Despite advancements, computational predictions are not always reliable. Factors like crystal packing, the arrangement of molecules within the crystal lattice, and specific interactions not fully accounted for by current methods can lead to inaccuracies [177,179,180,184]. Additionally, different computational methods have varying degrees of accuracy and applicability, depending on the system being studied [110,143]. While current methods predominantly focus on thermodynamic factors such as interaction energies, kinetic considerations such as crystal nucleation and growth rates are equally crucial but often overlooked.

Experimental validation remains essential to confirm computational predictions and accurately characterize the properties of cocrystals. However, synthesis challenges may prevent the facile synthesis of predicted cocrystals, complicating the validation process [183,186,187]. Furthermore, polymorphism adds another layer of complexity. Both APIs and cocrystals can exist in multiple crystal forms, and predicting the most stable and desirable form remains a significant challenge [180].

Despite these obstacles, ongoing research efforts aim to address these limitations. Initiatives include expanding databases with diverse cocrystal structures, improving data curation and standardization, and developing more interpretable ML models that elucidate the rationale behind predictions [110,177,188]. Researchers are also working to incorporate kinetic considerations into prediction methods and enhance experimental techniques for cocrystal synthesis and characterization [188,189].

Ultimately, by overcoming these challenges and refining both computational and experimental methods, cocrystal prediction can emerge as a potent tool for accelerating the discovery and development of novel materials with tailored functionalities. This convergence of efforts holds promise not only for advancing pharmaceutical development but also for other fields reliant on cocrystals, paving the way for new therapeutic agents, innovative materials, and potentially revolutionizing diverse technological applications [190].

## 4. Evaluation of the Methods and Regarding Tools Based on Three Selected Criteria

The programs listed in Section 2, Overview of Cocrystal Formation Prediction Methods, offer a range of functionalities from basic crystal structure generation to advanced quantum mechanical calculations and optimization techniques, catering to different needs in cocrystal formation prediction. The choice of software depends on factors like the specific needs of the research, the desired level of accuracy, and budget constraints. Some commercial software might offer more user-friendly interfaces and integrated workflows, while open-source options provide greater flexibility for customization.

### 4.1. Evaluation Criteria

Here is a breakdown of key criteria for evaluating cocrystal prediction tools in cocrystal discovery (Figure 2).

Efficiency is a key consideration in cocrystal formation prediction, as it directly impacts the practicality and speed of the research process. This criterion evaluates the accuracy and reliability of the predictions generated by each method, ensuring that the results are dependable and can be confidently used for experimental validation. Efficient methods also excel at identifying improbable coformers and prioritizing promising candidates by ranking them, streamlining the prediction process.

Cost-effectiveness is a critical consideration for researchers, particularly those with limited budgets. Efficient research is determined by a balance of time and financial resources. Time factors such as computation time, infrastructure requirements, software tools with employed algorithms, and data availability significantly impact productivity. On the budget side, perpetual and periodic licensing fees, hardware costs, and database expenses must be considered. By optimizing the use of these resources, researchers can ensure that their methods are not only financially available but also time-efficient, maximizing output while minimizing costs, allowing researchers to allocate their resources effectively.

User-friendliness in computational tools is largely defined by the interplay between theoretical knowledge and the learning curve associated with their use. It refers to how easy it is to learn and utilize the software, a crucial factor for experimental researchers who may not have extensive computational backgrounds. Researchers need to consider how much and how deep their theoretical knowledge of computational chemistry and coding is necessary to effectively navigate these tools for predicting cocrystal formation. However, the learning curve is significantly influenced by the quality of the graphical user interface (GUI), where a user-friendly interface with clear menus and intuitive functionality becomes essential, especially for those new to cocrystal prediction. Well-designed GUIs and minimal coding requirements enhance accessibility, allowing researchers to quickly become proficient and minimizing the time spent on training and troubleshooting. The time and effort required to learn the software effectively should also be taken into account. Comprehensive user guides, tutorials, and online resources can significantly reduce the learning curve, making the software more approachable. Furthermore, an active user community and readily available technical support are invaluable for troubleshooting issues and learning best practices. Access to forums or discussion boards where users can share experiences and seek assistance can provide significant advantages, fostering a supportive environment that enhances the overall usability of the software and ultimately contributes to more effective research outcomes.

### 4.2. Evaluation of the Selected Methods and Tools

The following subsections will analyze the methods based on three key criteria, namely, user-friendliness, efficiency, and cost-effectiveness. The focus will be on their usefulness for an experimental researcher in the workflow of rational cocrystal design. Table 1 summarizes the methods along with the associated programs/tools, briefly describing their ease of use, learning curve, accuracy and reliability, time required, initial cost, and hardware requirements.

#### 4.2.1. Quantum Mechanical Methods for Cocrystal Prediction: A Powerful Approach with Limitations

QM methods offer exceptional accuracy in cocrystal prediction due to their ability to model electronic structures and intermolecular interactions at a fundamental level. DFT methods excel in providing highly accurate electronic structure calculations and interaction energies [25]. This accuracy is crucial for understanding the non-covalent interactions, like hydrogen bonding, that are critical for cocrystal stability. The quantum mechanical foundation of DFT methods ensures precise predictions of these interactions, offering a significant advantage in cocrystal design. However, this high level of accuracy comes at a cost of computational intensity. Calculations can be time-consuming and resource-intensive, requiring access to powerful computers or high-performance computing clusters. Furthermore, DFT methods and MEP calculations performed with QM software are known for their complex setups and steep learning curves. They demand extensive knowledge of quantum mechanics and computational chemistry for effective utilization. This significantly limits their accessibility to researchers without a strong computational background. Many QM software packages offer extensive user guides, tutorials, and online communities, whose availability can significantly ease the learning process for researchers with a basic understanding of quantum mechanics and computational chemistry. While free, open-source options exist, commercial software can be costly. Therefore, QM methods are often best suited for researchers with advanced computational expertise or in collaboration with computational scientists.

#### 4.2.2. Molecular Docking: Accessible Powerhouse for Initial Cocrystal Screening

AutoDock [44] and Vina [45] are renowned for their accessibility and ease of use. They feature a well-documented GUI through AutoDockTools (ADTs) [191], which simplifies the preparation of input files and visualization of results. The availability of extensive tutorials and community support further enhances their user-friendliness, making them suitable for researchers with varying levels of computational expertise. AutoDock is efficient in predicting binding affinities and poses for cocrystal candidates. It uses a Lamarckian genetic algorithm and empirical free energy scoring functions to evaluate interactions. While it provides reliable results, the computational time can be significant, especially for larger systems. Both AutoDock and ADT are open-source and freely available, making them highly cost-effective. Its minimal hardware requirements and no licensing fees make it an attractive option for academic and budget-constrained research settings.

GOLD [46] is appreciated for its robust graphical user interface (GUI) and intuitive workflow. It offers detailed documentation and support resources, including user guides and forums. The interactive nature of the software allows users to easily adjust docking parameters and visualize docking poses, contributing to its high user-friendliness. GOLD excels in efficiency due to its genetic algorithm optimization, which is particularly effective in exploring the conformational space of API–coformer pairs. It provides highly accurate predictions of binding modes and affinities with reasonable computational times. The efficiency of GOLD makes it a preferred choice for high-throughput docking studies. GOLD is commercial software with licensing fees, which can be a significant cost factor. However, its efficiency and accuracy often justify the investment for researchers requiring reliable and high-throughput docking capabilities. The cost of GOLD is balanced by its robust performance and extensive support.

DOCK [49], while powerful, is less user-friendly compared to AutoDock and GOLD. It primarily operates through command-line interfaces, which can be challenging for users unfamiliar with such environments. Despite its comprehensive documentation, the steep learning curve associated with setting up and executing docking simulations can be a barrier for new users. DOCK is highly efficient in screening large libraries of compounds. It employs a grid-based approach to evaluate potential binding sites, making it faster for initial screenings. DOCK, like AutoDock, is an open-source tool, contributing to its cost-effectiveness. It requires minimal investment in software but may necessitate additional resources for computational power, particularly when screening large compound libraries.

Glide [48], part of the Schrödinger suite, is highly user-friendly, featuring an intuitive GUI that integrates seamlessly with other Schrödinger tools. The software provides step-by-step wizards for docking setup and analysis, along with extensive support resources. The design of Glide prioritizes user experience, making it accessible to researchers at all skill levels. Glide is noted for its exceptional efficiency and accuracy. It employs advanced algorithms such as the GlideScore scoring function and the hierarchical refinement of docking poses. Glide consistently delivers high-quality predictions with relatively short computational times, making it one of the most efficient docking tools available. Glide is a commercial product with substantial licensing fees. The cost can be a barrier for some users, but the integration of the software within the Schrödinger suite and its superior performance often offset the investment. Institutions with the budget for premium software will find the comprehensive capabilities of Glide and support services valuable.

Molecular docking methods are widely employed in the prediction of cocrystal formation due to their ability to simulate the binding interactions between an API and a coformer. Docking programs are not ideal for directly predicting the final crystal structure of a cocrystal. They excel at a different stage of the cocrystal prediction process, that is, identifying potential binding modes and interactions between the API molecule and candidate coformers. Understanding docked positions and scoring results requires a combination of visualization skills, knowledge of molecular interactions, and familiarity with the specific scoring functions of the docking software. Noted here is that docking programs are primarily designed for studying protein–ligand interactions. Therefore, their application in predicting cocrystals may not be straightforward for new users unfamiliar with the software. Additionally, these algorithms may inadequately describe API–coformer interactions.

#### 4.2.3. Molecular Modeling for Studying Dynamics Within a Known Cocrystal Structure

Molecular dynamics simulations using software like GROMACS, AMBER, and LAMMPS offer exceptional accuracy in studying the dynamic behavior of molecules within a known cocrystal structure and can provide valuable insights into intermolecular interactions and ligand mobility within the crystal lattice. However, their direct application for predicting new cocrystal formations is limited. The vast number of potential configurations and the computationally demanding nature of long simulations make exhaustive exploration through these methods highly time-consuming. Furthermore, a strong background in computational chemistry and programming to use MDs for cocrystal simulations is needed. A solid understanding of molecular mechanics force fields is crucial, and choosing an appropriate force field significantly impacts the accuracy of the simulation. Thus, researchers need to be able to select appropriate force fields for the specific molecules in the cocrystal and interpret the resulting data from the simulation. The simulation setup defines various parameters like temperature, pressure, simulation time step, and initial atomic positions. Data analysis extracts relevant information from the generated large amount of data about atomic positions, velocities, and energies over time. Therefore, despite the existing user-friendly interfaces (as noted here that some level of user-friendliness means, for example, a limited GUI and power and flexibility through scripting languages), a strong foundation in computational chemistry and programming remains essential for effectively utilizing them in cocrystal simulations.

#### 4.2.4. Crystal Structure Prediction: Solutions for Varying Skill Levels Catered to by Different Tools

Crystal structure prediction, though potentially aided by user-friendly software, generally demands a strong background in both computational chemistry and crystal structure representation. Understanding the forces between molecules and selecting appropriate computational methods necessitate knowledge of computational chemistry. Analyzing the generated data on potential energies and atomic arrangements requires interpreting it within the framework of this field. Crystal structure representation expertise is equally important. Knowledge of space groups and crystallographic data formats allows researchers to decipher how molecules pack together and visualize the predicted structures effectively. While the specific depth of knowledge may vary based on the software and system complexity, a solid foundation in both areas is crucial for successful crystal structure prediction.

CALYPSO [61], a freely available software for academic users, caters to researchers comfortable with command-line interfaces. Despite the lack of a GUI, CALYPSO offers comprehensive documentation, user guides, and manuals to bridge the gap. An active forum further enhances its usability by providing a platform for users to share experiences, troubleshoot issues, and learn best practices from the community. One key advantage of CALYPSO is its ability to interface with several major computational chemistry and materials science codes, allowing researchers to seamlessly integrate it into their existing workflows.

PyXtal [72], a free Python package, empowers researchers to predict crystal structures. It transcends a basic command-line tool by leveraging Python scripting for customization and automation. Users can build crystals from scratch or modify existing ones, defining parameters like space group, cell dimensions, and even partial atomic occupancy. The iterative algorithm of PyXtal enforces packing rules to prevent unrealistic atomic proximity and attempts multiple structure generations. By identifying the lowest-energy form from these trials, PyXtal offers a valuable tool for computational materials science, potentially predicting stable phases without needing experiments. Despite its efficiency, due to the moderate documentation and the lack of GUI, PyXtal is not an attractive solution for experimental researchers.

UPACK [75], despite being a valuable tool for crystal structure prediction, might not be ideal for a non-computational chemist due to its complexity. It lacks a user-friendly interface, requiring knowledge of command-line scripting for operation. There is no detailed documentation, only a webpage, which itself is targeted towards specialists, referencing advanced concepts like force fields and space groups. UPACK generates a vast number of hypothetical crystal structures, requiring expertise in interpreting and selecting the most relevant ones. Furthermore, the software utilizes a model for electrostatic interactions and repulsion–dispersion forces, demanding a background in these areas. While UPACK offers a valuable tool for crystal structure exploration, its usage seems best suited for researchers with a strong foundation in computational chemistry.

GULP [63], a free and open-source software package for academics, caters to researchers comfortable with scripting and computational workflows. While graphical user interfaces are accessible through commercial software suites like BIOVIA Materials Studio, GULP itself is a command-line-driven tool. To compensate for the lack of dedicated support, GULP offers extensive resources such as manuals, FAQs, examples, and a community forum. However, it is important to note that GULP is provided “as is”, meaning users are responsible for troubleshooting and resolving issues independently. Limited support can be obtained by contacting the developers, but a commercial license with dedicated support is available through Biovia. Overall, GULP’s free availability and extensive resources make it a powerful tool for researchers with a strong background in computational chemistry, but its command-line interface and lack of built-in support may pose challenges for beginners.

XtalOpt [65], a freely available software suite, offers a user-friendly GUI for researchers in the field of crystal structure prediction. This software caters particularly to those new to the field, thanks to its clear and comprehensive user guides and documentation. While XtalOpt excels at generating a vast number of candidate crystal structures through its evolutionary algorithm, it relies on external software packages for the final geometry optimization step. Notably, XtalOpt exhibits strong interoperability with established computational chemistry codes such as VASP, GULP, and CASTEP. This compatibility allows researchers to seamlessly integrate the structure generation capabilities of XtalOpt into their existing workflows for complete crystal structure prediction.

CrystalMaker software positions itself as a user-friendly and comprehensive solution for crystal and molecular structure modeling and diffraction studies [77]. Unlike some competitors, CrystalMaker boasts a GUI for easy navigation and intuitive use. This can be particularly beneficial for researchers who are new to crystal structure analysis. Beyond user-friendliness, CrystalMaker offers a full suite of functionalities, encompassing everything from building and manipulating crystal structures to visualizing and analyzing diffraction patterns. A key highlight is the Packing Explorer, specifically designed to assist in the creation of novel crystal structures, a valuable tool for material discovery. Additionally, CrystalMaker brings accessible energy modeling and lattice dynamics capabilities to personal computers, empowering researchers to explore these aspects without the need for high-performance computing resources. For those seeking support, CrystalMaker offers “First-Class Support”, ensuring assistance throughout the software usage process. While CrystalMaker is not free, it provides various pricing options and license solutions to cater to individual and institutional needs. This combination of user-friendly interface, comprehensive functionality, and dedicated support makes CrystalMaker a compelling choice for researchers in materials science and crystallography without strong computational chemistry and programing knowledge.

CrystalExplorer [78] stands out as a powerful software suite designed for the investigation of intermolecular interactions within molecular crystals. Its user-friendly GUI simplifies navigation and fosters accessibility for researchers with varying computational backgrounds. CrystalExplorer offers a comprehensive suite of modeling tools, empowering users to not only visualize but also analyze various aspects of molecular crystals. These tools likely encompass functionalities for constructing crystal structures, manipulating individual molecules within the crystal lattice, and exploring different packing arrangements. Furthermore, CrystalExplorer provides valuable visualization tools for intermolecular interactions. This includes the generation of the Hirshfeld surface, a graphical representation of intermolecular contacts, along with its corresponding two-dimensional fingerprint, which offers a quantitative representation of these interactions. The commitment of CrystalExplorer to user support is evident through its extensive documentation, including FAQs and a user manual, which guides users through its functionalities. Additionally, a dedicated support page allows researchers to seek assistance with any issues they encounter. Finally, CrystalExplorer offers flexible licensing options, ensuring affordability and accessibility for researchers from both academic and industrial settings.

#### 4.2.5. COSMO-RS: A Powerful but Potentially Pricey Tool for Cocrystal Prediction

Both COSMOtherm [85] and COSMOquick [84] offer GUIs designed to be user-friendly. COSMOquick is specifically designed for rapid screening with a streamlined interface for quick calculations, making it ideal for efficiently screening large coformer libraries. Some understanding of COSMO-RS theory and thermodynamic concepts is beneficial for interpreting results effectively. Users with a background in chemistry or chemical engineering will likely find it easier to grasp the underlying principles. Tutorials and documentation provided by BIOVIA can further aid in learning the software functionalities. Both COSMOtherm and COSMOquick leverage the COSMO-RS model, known for its good accuracy in predicting thermodynamic properties like excess enthalpy of mixing and solubility, which are valuable for cocrystal prediction. COSMOquick is designed for rapid screening of potential coformers based on their interaction favorability with the API. COSMOtherm offers a more detailed analysis of shortlisted candidates, which can be more time-consuming depending on the desired level of detail and system complexity. COSMOquick focuses on user-friendliness and rapid screening, making it a good initial step in a cocrystal discovery workflow. In contrast, COSMOtherm provides a more in-depth analysis of thermodynamic properties, offering valuable insights for researchers. Both COSMOtherm and COSMOquick are commercial software products from BIOVIA and require paid licenses. The computational demands depend on the size and complexity of the molecules being studied. While both software programs can likely run on personal computers, larger systems or high-level calculations might benefit from more powerful workstations.

COSMO-RS-ADF [88] leverages ADF, a powerful quantum chemistry program, and offers a user interface that integrates COSMO-RS setup. Tutorials and documentation are available to assist users, but a good understanding of COSMO-RS theory, quantum mechanics, and ADF software usage is beneficial. The learning curve can be reduced by utilizing these resources. COSMO-RS-ADF combines quantum chemical calculations with the COSMO-RS model, leading to high accuracy in predicting thermodynamic properties relevant to cocrystal prediction, such as solubility. It is efficient for routine calculations on smaller molecules; however, for complex systems or large-scale screening, the calculations can become more time-consuming. COSMO-RS-ADF is part of the commercial ADF software suite [87,89], requiring a paid license and powerful computers.

The openCOSMO-RS [91] offers Python and C++ codebases, requiring some programming knowledge for setup and execution. Compared to commercial software with graphical interfaces, it has a steeper learning curve. A good understanding of COSMO-RS theory and experience with scientific computing is necessary to effectively use openCOSMO-RS. Familiarity with Python or C++ programming, as well as experience with scientific computing libraries, is beneficial for advanced users. Tutorials and documentation are available to assist users, but some background knowledge is helpful. The openCOSMO-RS can accurately calculate thermodynamic properties like solubility and excess enthalpy of mixing, relevant for cocrystal prediction, and offers efficient screening capabilities compared to full atomistic simulations. The exact time required depends on the system size, complexity of calculations, and computing resources available. As open-source software, openCOSMO-RS is free to use and requires moderate computational resources compared to complex simulations. While it can be run on personal computers, larger calculations might benefit from high-performance computing clusters.

In general, the accuracy of COSMO-RS is considered to be very good and performs well in predicting cocrystals compared with other methods [110,137,143].

#### 4.2.6. Hansen Solubility Parameters: Fast and Easy Screening for Initial Compatibility, Despite Limitations

HSP concepts are relatively straightforward, involving the calculation of parameters from group contributions, but using them effectively requires an understanding of solubility and intermolecular interactions [92]. HSP is a semi-empirical method, and its accuracy depends on the quality and completeness of the data used for parameter estimation. The underlying theory of HSP is freely available in the scientific literature, and the method itself does not require specific hardware. However, the commercially available HSPiP software [94], which simplifies the process, requires a paid license. The HSPiP software offers a user-friendly interface for calculations and visualization, which facilitates the process compared to manual calculation. However, a basic understanding of HSP theory and interpretation is necessary, and some training or experience with the program might be needed. HSPiP, as a Windows-based program, requires minimal hardware beyond a standard computer. Despite the lower reliability of the HSP concept compared to COSMO-RS calculations in cocrystal predictions [137,145], HSP can still serve as a quick and cost-effective starting point in the workflow of rational cocrystal design [93].

#### 4.2.7. Machine Learning for Cocrystal Prediction: A Promising but Evolving Field

ML tools for cocrystal prediction vary in their user-friendliness and the level of programming expertise required. Beginner-friendly options like scikit-learn [98] and WEKA [101,102] offer lower barriers to entry for users with basic programming knowledge and familiarity with ML concepts. These tools provide tutorials and documentation to help users get started with basic cocrystal prediction tasks. However, building custom models requires significant expertise in data science, ML algorithms, and coding skills, which presents a high learning curve. Even with user-friendly tools, understanding ML concepts and data analysis is beneficial for interpreting results and recognizing limitations in predictions. The accuracy of ML models relies heavily on the quality and quantity of training data, and limited data on known cocrystals can restrict the ability of a model to generalize to new systems [110,162,188,192,193]. Additionally, the “black box” nature of ML models can make it difficult to understand the rationale behind their predictions, limiting interpretability and trust [185]. Building models is time-consuming, involving data gathering, training, and optimization. However, using existing models with familiar software can be relatively quick once the user is acquainted with the process. Hardware requirements vary, with moderate computational resources typically being sufficient.

ML is a promising field that can significantly aid in prioritizing potential cocrystal formers for experimental validation, thereby saving time and resources [188,192]. The models can identify patterns in existing data that might not be apparent through traditional methods, helping researchers focus on promising cocrystal candidates with desired properties.

#### 4.2.8. Traditional QSAR Approaches: Complex Interaction Limit the Modeling Cocrystal Formation

The QSAR method offers moderate to high ease of use, but it requires a solid understanding of chemical properties, model development, and result interpretation. The learning curve is steep due to the need to grasp underlying QSAR principles, data preparation, and statistical analysis. Developing QSAR models is time-consuming, involving extensive data collection, curation, model building, and validation. However, the method itself is accessible, with various free and open-source software available for model development. Tutorials and documentation are available, which can help ease the learning process, although a background in QSAR concepts is beneficial.

The QSAR Toolbox, with its moderate ease of use, provides a user-friendly interface with pre-built models and functionalities. While some knowledge of QSAR is helpful, the availability of tutorials, manuals, webinars, and a helpdesk makes it accessible. Prediction time using existing models with the QSAR Toolbox is efficient, and the software is freely available, offering a good balance between ease of use and functionality for those familiar with QSAR concepts.

The KNIME Analytics Platform offers a moderate to high ease of use with its visual programming interface. It requires some familiarity with data analysis and potentially scripting for advanced workflows. The learning curve is moderate to high; while the visual interface lowers the entry barrier, advanced functionalities might necessitate scripting knowledge or data science expertise. KNIME workflows for QSAR analysis can be efficient for established workflows, but building complex workflows can be time-consuming. The core version of KNIME is free, with paid options for additional features, providing a flexible platform for custom workflows that integrate QSAR models with other data analysis tools, although it demands more technical knowledge compared to the QSAR Toolbox.

Both tools are valuable for researchers predicting chemical properties or activities based on structure. Hardware requirements also vary with the complexity of the QSAR model and tools used, but generally, moderate computational resources are sufficient.

In general, the accuracy and reliability of QSAR models vary depending on the specific model, data quality, and chosen endpoints. In the case of cocrystal prediction, QSAR models have low accuracy and reliability [193]. Due to the multifaceted nature of intermolecular interactions governing cocrystal formation, including hydrogen bonding, van der Waals forces, and π–π stacking, a complex network of forces dictates successful cocrystallization. This complexity poses a challenge for traditional QSAR methods, which typically focus on simpler, more direct relationships between chemical structure and a singular biological activity. Consequently, capturing the intricate interplay of forces involved in cocrystal formation may be beyond the capabilities of standalone QSAR models, potentially limiting their effectiveness in this specific application. While QSAR remains a valuable tool for understanding various aspects of molecular behavior, its application to cocrystal prediction might necessitate additional considerations or complementary methods to account for the complex interplay of forces at play. However, novel approaches combining different descriptors can significantly improve the accuracy of QSAR cocrystal prediction, addressing some of the complexities involved [156].

#### 4.2.9. Database and Knowledge-Based Methods: A User-Friendly and Efficient Starting Point for Cocrystal Discovery

Public databases like ZINC [14,15], PubChem [16], and ChemSpider [116] are extremely user-friendly, featuring mostly web interfaces with search filters that require a very low learning curve. These platforms are designed for ease of use, necessitating only a basic understanding of search terms, thanks to their intuitive interfaces. The CCDC software suite [47], which includes tools like ConQuest [111], Mercury [112], and the CSD Python API [113], has a moderate level of ease of use and requires some understanding of crystallography concepts. Tutorials and documentation are available to assist users, leading to a moderate learning curve. DrugBank [115] also has a moderate ease of use with a user-friendly interface, though familiarity with biological data is beneficial. The learning curve for DrugBank is moderate, as it helps to have knowledge of bioinformatics and cheminformatics concepts.

The accuracy and reliability of these tools depend significantly on the specific tool and the quality and comprehensiveness of the underlying data. For virtual screening methods, the accuracy and reliability hinge on the quality of the search criteria and the methods chosen, such as shape/size versus functional group matching. While virtual screening provides an initial filtering based on user-defined criteria, it is not a definitive prediction method, serving rather as a preliminary step in the drug discovery process.

## 5. Comparative Analysis and Recommendations for Experimental Researchers

**Figure 5:**
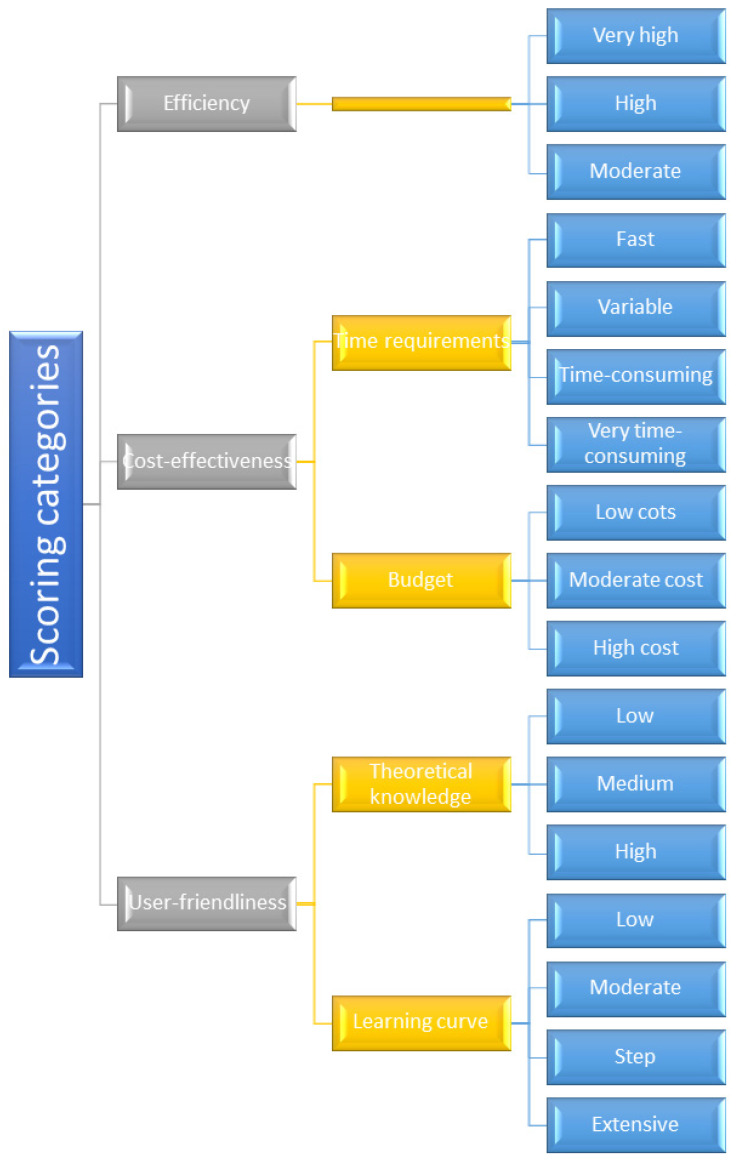
The categories of our scoring model.

Creating an objective scoring model to rank in silico screening methods for cocrystal formation predictions based on our three criteria—efficiency, cost-effectiveness, and user-friendliness—is nearly impossible. Although we have made efforts to establish a scoring model (Figure 5) that minimizes subjectivity, some intuitiveness remains.

Efficiency is the most complex criterion in our evaluation. Based on our literature review, we conclude that each method is capable of predicting the formation of cocrystals between the selected API and the investigated coformer. To establish a clearer ranking, we have categorized the methods into three groups based on their efficiency: very high, high, and moderate, corresponding to scores of 3, 2, and 1 points, respectively.

The score of cost-effectiveness is defined as the average of the budget and time requirements points. Considering the budget, low cost (free, open-source tools compatible with standard computers), moderate cost (free, open-source tools requiring high-performance computers, or licensed software with standard computers), and high cost (licensed software requiring high-performance computers) scored by 3, 2, and 1 points, respectively. When the time requirements of the in silico screening are fast, variable, time-consuming, or highly time-consuming, earn 4, 3, 2, or 1 point, respectively.

The average of the theoretical knowledge and learning curve points results in the user-friendliness score. Low, medium, and high computational chemistry and coding knowledge receives 3, 2, and 1 point. While low, moderate, step, and extensive learning curves get 4, 3, 2, and 1 point.

**Figure 6:**
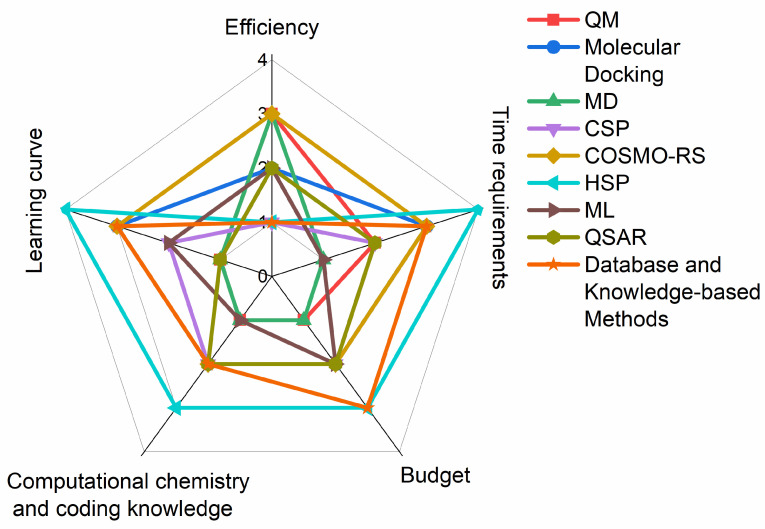
In silico screening method comparison across scoring categories.

**Figure 7:**
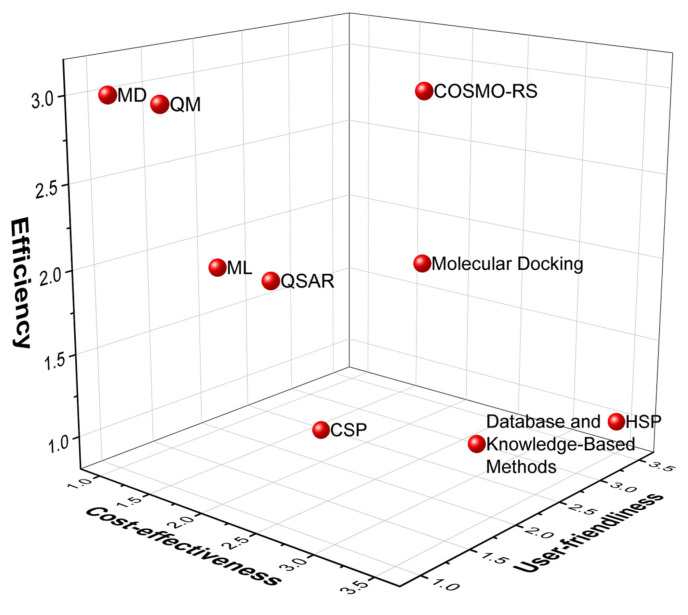
Clusters of in silico screening methods with similar characteristics.

Figure 6 compares the performance of different in silico methods for cocrystal formation prediction across the scoring categories of the key criteria, such as efficiency, cost-effectiveness, and user-friendliness. While Figure 7 identifies clusters of methods with similar characteristics.

Although both QM and MD methods offer high accuracy in predicting stable cocrystal structures, they are not recommended for most experimental researchers due to several limitations, such as advanced knowledge requirements in quantum chemistry and programming, computational intensity, including extended periods of simulation runs, and supercomputing resources; furthermore, the associated software licenses and computational resources can be expensive.

Developing reliable QSAR models requires significant time investment, and the software’s user-friendliness can be moderate. CSP offers a slightly more user-friendly environment compared to QSAR, but the cost is generally higher. Additionally, refinement of predicted structures often necessitates advanced QM methods. Docking programs are user-friendly, but they are not specifically designed for API–coformer interactions. This can lead to questionable reliability for cocrystal prediction. While docking offers a cost advantage over QM methods, its limitations make it less suitable for this specific application.

The ML models hold significant promise for cocrystal prediction. Several free programs exist for model building; however, these models can be challenging to use due to their “black-box” nature. Programming skills (e.g., Python) are beneficial for effective utilization. With appropriate data training, ML models can deliver accurate predictions. With continued development, the future looks bright for user-friendly and accurate ML-based cocrystal prediction tools.

Database and Knowledge-Based Methods offers a cost-effective approach, with many databases freely accessible. However, the reliability of data in these databases can vary, and manual data collection can be time-consuming. Utilizing available software, such as Mercury, can facilitate efficient screening in a short time.

The COSMO-RS method takes the top spot due to its exceptional accuracy in predicting cocrystal formation. User-friendly software readily available in commercial markets further enhances its appeal. While the HSP method with HSPiP software provides an excellent entry point for researchers new to cocrystal design. This popular method is highly user-friendly, with readily available software at a low cost. While its predictions may not be the most detailed compared to other techniques, the HSP method offers a rapid and easy-to-understand approach for initial cocrystal exploration.

**Figure 8:**
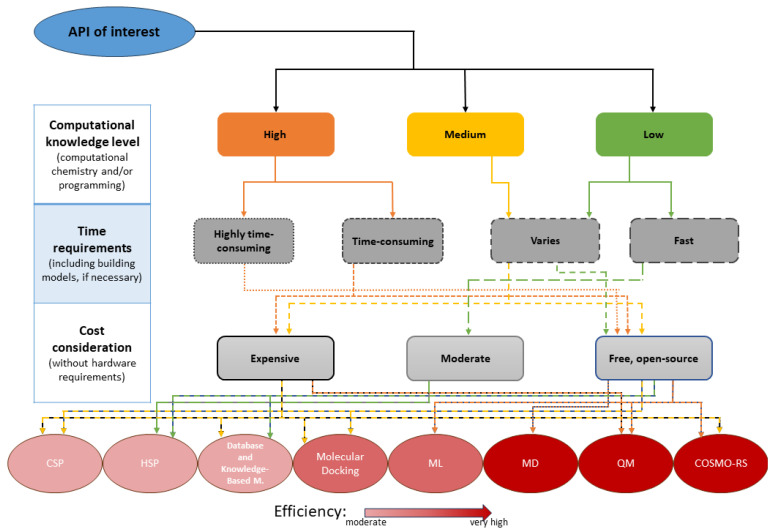
The flowchart to facilitate the decision-making process to choose the method for cocrystal formation prediction.

This ranking provides a framework for researchers to select the most suitable cocrystal prediction method based on their individual needs and resources. The choice of the methods depends on the specific needs of the researcher (Figure 8). If efficiency is the primary concern, QM or MD might be preferred. If cost-effectiveness and user-friendliness are more important, then HSP and COSMO-RS with GUI could be suitable.

## 6. Conclusions

The prediction of cocrystal formations is a crucial step in rational cocrystal design. It utilizes a multifaceted approach, combining computational and theoretical methods to forecast the stability of cocrystal formation between an API and a coformer molecule. This predictive capability empowers researchers to prioritize promising candidates for experimental synthesis. This significantly enhances the efficiency of drug development processes by focusing efforts on candidates with a higher likelihood of success.

While various methods exist for cocrystal prediction, user-friendliness, efficiency, and cost-effectiveness are crucial factors in selecting the most suitable approach. Any method can effectively predict the potential for cocrystal formation if applied correctly, while none will yield satisfactory results if misused. However, if the primary objective is to identify the molecules most likely to form a cocrystal, the simplest approach—when configured appropriately—is often the best choice. By starting with more straightforward methods and gradually advancing to more complex ones as needed, researchers can efficiently identify promising cocrystal systems and optimize their properties.

Recommendations for researchers are as follows:

First Choice—HSP with HSPiP: HSP combined with HSPiP is our top recommendation due to its ease of use [93], fast screening, and reasonable cost. With careful parameterization, its accuracy can be significantly enhanced. The method only provides a formation prediction without providing any cocrystal structural insights.

Second Choice—COSMO-RS with GUI: For researchers seeking a balance between computational effort and ease of use, COSMO-RS with a GUI is a solid choice [82]. The GUI simplifies interaction, making it accessible even for users with limited computational expertise.

Third Choice—Virtual Screening in CSD with Mercury: Virtual screening in the CSD using Mercury offers a powerful advantage in cocrystal formation prediction. Researchers can efficiently search a vast database of crystal structures, identifying potential cocrystal partners with similar packing motifs and favorable intermolecular interactions [114]. It provides a formation probability and plausible structure, which is a valuable starting point for experimental cocrystal design.

It is important to acknowledge the inherent subjectivity in method selection. Individual user experience and familiarity with computational tools can significantly influence the perceived effectiveness and ease of use. Ultimately, the choice of prediction method should be based on a careful consideration of specific project needs, available resources, and user expertise.

## Figures and Tables

**Table 1:** Evaluation of cocrystal prediction methods and related programs and tools.

Method	Program/Tools	Ease of Use	Learning Curve	Accuracy and Reliability	Time Required	Initial Cost	Hardware Requirements
Quantum Mechanical Methods	Gaussian VASP Quantum ESPRESSO Schrödinger suite (Jaguar and MacroModel) Octopus	Requires significant expertise in quantum mechanics and computational chemistry.	Steep learning curve; extensive training needed.	Highly accurate and reliable for electronic structure calculations.	Computationally expensive and time-consuming calculations.	Cost varies; free open-source options are available, but commercial software can be expensive	Requires access to powerful computers or high-performance computing clusters.
Molecular Docking	AutoDock GOLD DOCK	Reasonably accessible with good training	Moderate learning curve; basic understanding of molecular interactions required	Effective for initial screening and predicting binding affinities/poses	Varies depending on system size and complexity; generally faster than QM methods	Variable; free and commercial options available	Modestly powerful computers; GPUs can improve speed (optional)
Molecular Dynamics	GROMACS AMBER LAMMPS	Moderately complex	Steep learning curve; requires understanding of molecular mechanics and scripting	Highly accurate	Highly time-consuming for exhaustive cocrystal exploration	Several free options are available	Requires powerful computers or high-performance computing clusters
Crystal Structure Prediction	CALYPSO XtalOpt PyXtal UPACK GULP CrystalMaker CrystalExplorer	Moderately complex and complex (requires scripting)	Moderate or steep	Varies	Varies (depends on system complexity and desired accuracy and can be time-consuming)	Variable; free and commercial options available	Powerful computer recommended
COSMO-RS	BIOVIA COSMO-RS (COSMOquick, COSMOtherm) COSMO-RS-ADF-openCOSMO-RS	Variable; user-friendly (menus/interfaces); Moderate to high (depending on experience in QM calculations)	From moderate training (some background helpful) to a step learning curve	Potentially high for thermodynamic properties and solubility prediction	Can be efficient for screening and routine calculations, less time-consuming than full simulations	Variable; commercial options, open-source alternative	Variable; moderate computational resources; high; requires access to powerful computers
Hansen Solubility Parameters	HSPiP	User-friendly (menus/interfaces)	Moderate training	Accuracy depends on the data quality and limitations of the HSP method	Efficient for screening	HSP method (free), HSPiP (commercial software)	Minimal; HSPiP requires Windows PC
Machine Learning	scikit-learn TensorFlow PyTorch WEKA CrySPY	Depending on the specific tools, “black-box “, Experience in programming required	Steep	Depending on the quality and quantity of training data	Building models is time-consuming; the use of pre-trained models is more efficient	The most tools are free and open-source	Moderate computational resources
Quantitative Structure-Activity Relationship	KNIME Analytics Platform QSAR Toolbox	Moderate-high	Moderate-High (basic understanding of QSAR and depends on the desired level of expertise)	Low in cocrystal prediction	Can be time-consuming.	Free options and free core versions	Moderate computational resources, but varies depending on the complexity of the QSAR model and the chosen tools
Database and Knowledge-Based Methods	CCDC software suite (ConQuest, Mercury, CSD Python API) COD ZINC Database PubChem ChemSpider DrugBank	Varies depending on the specific tool. CCDC software requires some training, while ZINC and PubChem offer user-friendly interfaces.	The learning curve varies. CCDC software requires more understanding of crystallography and search criteria, while ZINC and PubChem are more intuitive.	Depends on search criteria, methods, and database quality	Efficient for screening, time depends on the screening strategy and tools	Variable; most options are free, but CCDC software is commercially available	Minimal (web browser) or standard computers

## Data Availability

No new data were created.
